# Establishing a Pharmacy-Based Pre-Exposure Prophylaxis Program for Young Women Who Sell Sex: Protocol for a Randomized Controlled Trial

**DOI:** 10.2196/74141

**Published:** 2025-12-03

**Authors:** Oppah Kuguyo, Helena Archer, Lance Azizi, Constancia Watadzaushe, Sharon Munhenzva, Kudzai Chidhanguro, Albert Takaruza, Sithembile Musemburi, Laura Packel, Jenny Liu, Primrose Matambanadzo, Frances M Cowan, Sandra McCoy, Euphemia L Sibanda

**Affiliations:** 1 Department of Sexual and Reproductive Health Centre for Sexual Health HIV/AIDS Research Zimbabwe Harare Zimbabwe; 2 School of Public Health University of California, Berkeley Berkeley, CA United States; 3 University of California, San Francisco San Francisco United States; 4 Liverpool School of Tropical Medicine Liverpool United Kingdom

**Keywords:** pre-exposure prophylaxis, female sex worker, key populations, HIV/AIDS, PrEP continuity, women who sell sex, differentiated service delivery

## Abstract

**Background:**

Sex workers (SWs) are at risk of acquiring HIV infections. Despite this, SWs have low uptake of relevant preventive measures, such as HIV pre-exposure prophylaxis (PrEP). Community-based programs that make PrEP convenient and accessible have the potential to drive PrEP continuation and reduce HIV risk.

**Objective:**

This study aims to describe the co-development and piloting of the Together Optimizing PrEP Access in Zimbabwe (TOPAZ) intervention—where SWs have the opportunity to access PrEP refills through pharmacies in Zimbabwe.

**Methods:**

Using a participatory process, the TOPAZ intervention will be collaboratively developed with pharmacy owners and SWs. TOPAZ comprises the provision of safe and convenient spaces for PrEP refill pickups bundled with a gift voucher incentive at pharmacies. Formative research comprising focus group discussions and in-depth interviews with SWs, pharmacists, and stakeholders will inform the development of the preliminary model (aim 1). A cluster-randomized pilot trial will evaluate the impact of the PrEP-in-pharmacy intervention, compared to standard clinic-based pickup in the key populations program, on PrEP retention at 3, 6, 9, and 12 months after initiation (aim 2). The outcome of the pilot trial will be a comparison of PrEP retention at 6 months after initiation between study arms. We will also compare PrEP retention among SWs aged between 18 and 24 years at 6 months and among participants remaining active in sex work at 6 months. We will use the Generalized Estimation Equations model to generate risk differences and 95% CIs for PrEP retention in intervention versus comparison arms. Analysis will follow an intent-to-treat approach. Finally, a mixed methods study will be conducted to understand the potential for scaling TOPAZ for SWs; insights from this study will inform the design of a future effectiveness trial (aim 3). Proctor’s implementation outcomes, namely, acceptability, adoption, appropriateness, feasibility, fidelity, and penetration of the pharmacy-based PrEP refill program, will be evaluated by triangulation of program data and qualitative interviews.

**Results:**

This study began the enrollment of participants in March 2024. Data collection ended in May 2025. The final comprehensive analysis of the results will be conducted in February 2026 after all the data have been collected and reviewed comprehensively.

**Conclusions:**

This study will report on the feasibility, acceptability, and effectiveness of pharmacy-based PrEP refills to improve PrEP continuation among women who sell sex. If successful, this preliminary study will pave the way for a future effectiveness trial to evaluate this intervention more comprehensively.

**Trial Registration:**

ClinicalTrials.gov NCT06348069; https://clinicaltrials.gov/study/NCT06348069

**International Registered Report Identifier (IRRID):**

DERR1-10.2196/74141

## Introduction

### Background

Sex workers (SWs) are disproportionately affected by HIV and contribute a 4-fold higher HIV prevalence than adults in the general population [1]. The relative risk of acquiring HIV among SWs globally is 9 times higher than the general population, while in sub-Saharan Africa, the relative risk of HIV acquisition is 11 times higher in SWs than the general population [2]. Data from generalized HIV epidemics in sub-Saharan Africa reveal that SWs and their clients account for >30% of HIV infections and 17% of incident HIV infections [2,3]. In Zimbabwe, HIV prevalence among female SWs (FSWs) is >50%, and the annual estimated incidence is 4%, translating to a 10-fold higher incidence than women in the general population [4,5]. New entrants into sex work and young women who sell sex are at the highest risk of HIV infection [5-8]. For example, HIV prevalence increases sharply from 2% for an FSW aged 16 years to >50% for an FSW aged 24 years in Zimbabwe [4,6-9]. These alarming statistics underscore a need to strengthen the effective use of HIV prevention for FSW, particularly among younger FSW, both to protect them from HIV and to support broader HIV epidemic control efforts [10-12].

Oral pre-exposure prophylaxis (PrEP) has demonstrated high efficacy in clinical trials, preventing >92% of new HIV infections when taken as prescribed [13]. However, implementation in real-world contexts has been disappointing, with low uptake and suboptimal continuation. Thus, strengthening motivation, capability, and effective use of PrEP among SWs, including younger FSWs, has the potential to reduce the rate of new infections among both SWs and the general population more broadly [5]. Mathematical modeling data from South Africa anticipate that high uptake and continuity of PrEP could theoretically lead to a population-level reduction in HIV incidence by 40% [7,8].

Although PrEP uptake reduces HIV risk, effective PrEP use and continuation is very low among populations considered high risk, such as SWs [14-16]. Studies from Benin (45%), Rwanda (22%), and Senegal (27%) reported lower PrEP discontinuation within 12 months when compared to South Africa (91%) and Zimbabwe (86%) [16-21]. These differences underscore the need to identify barriers to PrEP continuation toward developing context-specific interventions.

A host of factors impede PrEP uptake and continuation among FSWs, but of relevance to this intervention are fear of stigma associated with PrEP and lack of convenient access [20,22]. The World Health Organization recommends differentiated service delivery to diversify PrEP delivery, overcome barriers to access, and strengthen PrEP continuation [23]. Developing interventions outside the “brick-and-mortar” health clinics, for example, private pharmacies, may decentralize PrEP services and improve access [24-28]. Pharmacies are proven community-based channels that advance self-care initiatives in sexual and reproductive health and may be tailored to deliver PrEP for individuals considered high risk. Developing pharmacy-based PrEP service delivery models for SWs could make PrEP more accessible and improve PrEP uptake and continuation.

Successful delivery of pharmacy-based PrEP is contingent on collaborative efforts between policy makers, communities, and pharmacists [14,15,29-35]. This study aims to explore whether providing SWs with the option of PrEP refill supported by HIV testing through community-based pharmacies is feasible, acceptable, and increases effective use of PrEP in Zimbabwe. Given that research shows that small, short-term incentives can also be used to increase habit formation and motivation to continue PrEP [36-38], our trial also includes provision of small incentives to motivate PrEP continuation. Our Together Optimizing PrEP Access in Zimbabwe (TOPAZ) study develops and tests this pharmacy-based PrEP intervention for FSWs in Zimbabwe.

### Study Aims

The overall goal of the TOPAZ study is to increase PrEP continuation among FSWs through a co-developed pharmacy-based intervention. Specific aims are as follows: aim 1 is to refine and operationalize pharmacy-based PrEP distribution, including an escalating incentive with FSWs and pharmacy owners to motivate PrEP continuation in FSWs; aim 2 is to determine whether the addition of pharmacy-based PrEP distribution increases PrEP retention among FSWs at 6 months compared to standard services; and aim 3 is to understand the PrEP-in-pharmacy program’s potential to scale and translate lessons learned into a future effectiveness study.

## Methods

### Overview

**Figure 1:**
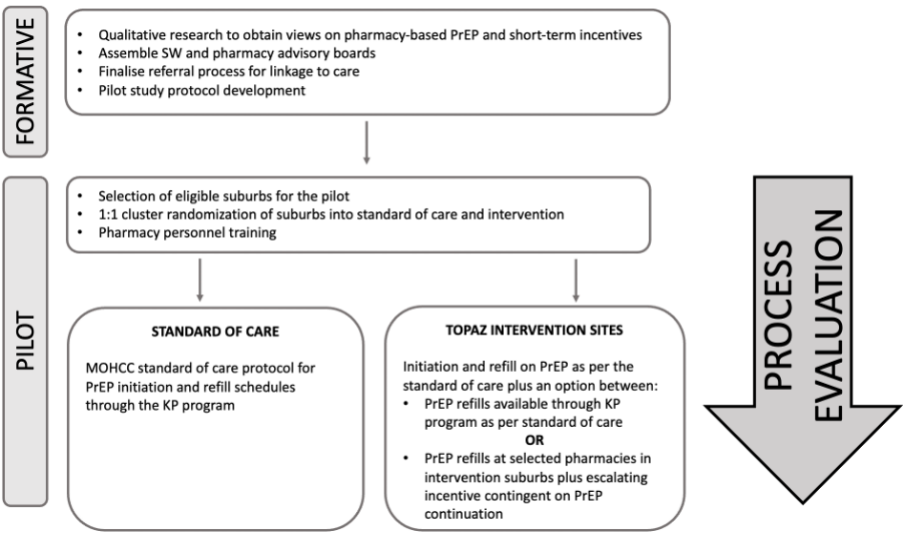
Overview of the Together Optimizing PrEP Access in Zimbabwe (TOPAZ) study design. KP: key populations; MoHCC: Ministry of Health and Child Care; PrEP: pre-exposure prophylaxis; SW: sex worker.

A mixed methods approach will be used to develop and pilot the PrEP-in-pharmacy refill intervention for SWs, as summarized in Figure 1. The PrEP-in-pharmacy refill intervention will be available to all SWs in their diversity, including male and transgender workers; however, this study will formally assess outcomes among FSWs. To achieve aim 1, formative research to co-develop the intervention with SWs and pharmacists will be conducted. To achieve aim 2, the intervention will be piloted through an existing key populations (KP) program. A 1:1 cluster-randomized control design will be used to allocate 8 suburbs to 4 intervention or 4 standard-of-care suburbs. To achieve aim 3, a process evaluation will be conducted using a mixed methods implementation science framework to determine the feasibility, acceptability, and scalability of the intervention and to optimize future versions of the program.

### Theoretical Model Informing the Implementation

**Figure 2:**
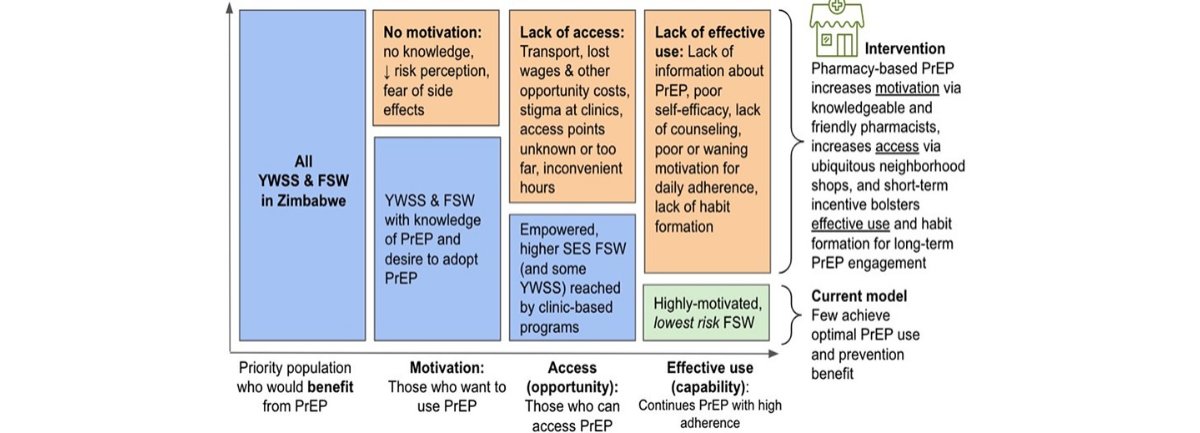
Application of the Capability Opportunity Motivation Behavior model in the current PrEP delivery for YWSS and FSWs in Zimbabwe and the contributions of the proposed intervention, which provides friendly, pharmacy-based PrEP distribution by informed pharmacists plus financial incentives (adapted from the study by Schaefer et al). FSW: female sex worker; PrEP: pre-exposure prophylaxis; SES: socioeconomic status; YWSS: young women who sell sex.

This research draws on 2 primary theoretical models of behavior change: the Capability Opportunity Motivation model of behaviour change and behavioural economics Capability Opportunity Motivation Behavior (COM-B) model of behavior change and behavioral economics (Figure 2 [39]) [37,38]. The pharmacy intervention will be designed to increase *motivation* through knowledgeable and friendly pharmacists plus the peer outreach and support services already provided by the existing KP program. In addition, small incentive gift vouchers that can be redeemed for pharmacy products offered to FSWs for collecting PrEP refills at pharmacies will also increase *motivation*. *Access* will be increased through PrEP provision at pharmacies, and *effective use* will be increased by combining pharmacy-based access with short-term, escalating incentives (gift vouchers) to encourage continued PrEP refills [38]. Underpinning the incentive component of the intervention is behavioral economic theory, which posits that using simple, scalable “nudges” (norms, commitments, and incentives) that capitalize on common biases can influence behavior [40]. This approach is particularly useful when applied to behaviors that are new (such as taking PrEP) or those that can be viewed as unimportant or inconvenient (such as collecting PrEP refills, especially due to present-biased preferences) [41]. Escalating incentives leverage the behavioral economics concept of *loss aversion*, increasing “losses” are faced each time a refill visit is missed, a simple yet powerful motivator to stay engaged with PrEP [42-44].

### Ethical Considerations

The TOPAZ study has obtained ethics approval from the national ethics review committee in Zimbabwe, the Medical Research Council of Zimbabwe (MRCZ/A/2988), and the University of California Berkeley Committee for Protection of Human Subjects (2022-11-15783). All study procedures will be conducted in accordance with the Declaration of Helsinki. All participants will be informed about the study and will provide consent to participate, as outlined in the following sections describing the various study activities. Participant data will be deidentified and stored in secure databases, with identifiable data, such as full names (only in the informed consent), stored separately. Adverse occurrences throughout the study will be recorded in a log that will be shared with the national ethics review board.

### Study Setting and Population

This intervention will be nested within the existing KP program, a nationally scaled program for SWs run by the Centre for Sexual Health, HIV, and AIDS Research Zimbabwe on behalf of the Ministry of Health and Child Care (MoHCC) and National AIDS Council. The program aims to reduce HIV acquisition and transmission via the provision of comprehensive sexual and reproductive health services at mobile and static clinics tailored for KP and supported by peer-led community engagement activities [5]. As of 2023, the KP program services include contraception, syndromic management of sexually transmitted infections, mental health screening and treatment, gender-based violence prevention and referral, HIV testing, initiation of antiretroviral therapy, HIV postexposure prophylaxis, and PrEP. The KP program operates mobile outreach clinics in 14 suburbs across Harare, from which 8 TOPAZ study suburbs were selected. Eligible suburbs needed to have ≥3 pharmacies that have a private area to implement the intervention and were willing to implement the intervention. Consequently, the PrEP-in-pharmacy intervention will be conducted in Glen Norah, Mufakose, Epworth, Glen View, Highfield, Kuwadzana, Mabvuku, and Kambuzuma.

### Pharmacy Selection Process

A mapping exercise will be conducted in the selected suburbs to identify pharmacies that meet the TOPAZ pharmacy criteria. A sensitization process will be used to assess the pharmacy owners’ interest in participating in the study. A total of 3 pharmacies per suburb will be shortlisted, and in instances where there are >3 qualifying pharmacies in a suburb, distance from a neighboring TOPAZ study pharmacy will be considered in the selection of the 3 participating pharmacies to ensure pharmacies are well spaced within the suburbs.

### Design

To achieve aim 1, a qualitative study will be conducted. Aim 2 will involve a cluster-randomized control trial, with suburbs as the unit of randomization. In total, 8 suburbs will be randomized to either standard of care, which is collecting PrEP services from the KP program (*comparison condition*), or standard-of-care KP program PrEP services *plus* the option of pharmacy-based PrEP refills, which includes a financial incentive of a gift voucher for pharmacy products (*intervention condition*). FSWs in the KP program who are newly initiated onto PrEP will be recruited into the TOPAZ study prospectively, and outcomes will be measured at 3, 6, 9, and 12 months after PrEP initiation. Aim 3 will use a mixed methods approach to determine the feasibility, acceptability, and scalability of the PrEP-in-pharmacy intervention.

### Aim 1: Formative Research

#### Overview

Formative research activities will be conducted to ensure that the proposed approach addresses barriers to PrEP retention on both the supply (pharmacy) and demand (SWs) sides. Two key stakeholder groups, the pharmacists and SWs, will be engaged to establish advisory boards. The SW advisory board will ensure that the final intervention design aligns with the needs and preferences of the SWs. The pharmacist advisory board will play a key role in operationalizing the PrEP-in-pharmacy intervention and ensuring it is integrated into routine business operations within pharmacies. Consultative meetings will be held monthly to develop the intervention process (Table 1).

**Table 1:** Qualitative approaches to be applied in the formative research phase of the Together Optimizing PrEP Access in Zimbabwe study implementation.

Group	Method	Interviews, n	Inclusion and exclusion parameters	Contribution
Pharmacy advisory board	Consultative meetings	15	Inclusion criteria Pharmacies that are officially registered Pharmacies located in Harare Available to participate in board meetings during the intervention	Inform intervention development from a pharmacy perspective
SW^a^ advisory board	Consultative meetings	5	Inclusion criteria Enrolled in the KP^b^ program Demonstrating knowledge about PrEP^c^ Residing in Harare Available to participate in board meetings over a 24-month period Exclusion criteria SW intending to move out of Harare during the intervention	Inform development of the intervention from an SW perspective
SW	Semistructured in-depth interview	20	Inclusion criteria Aged >16 years Exchanged sex for money or gifts in the last 6 months^d^	Barriers to PrEP retention Mitigating PrEP-related stigma Using pharmacies for PrEP refills Procedures for using and reporting HIV test results Referral services How to create a welcoming environment
Pharmacy personnel^e^	Semistructured in-depth interview	6	Inclusion criteria Aged >18 years Own or work at a pharmacy in Harare	Perspectives of engaging FSWs^f^ in their shops Attitudes and beliefs about SWs Perceived roles in community-based health provision Relationship with referral facilities and staff Views on the viability of pharmacies as distribution sites for daily oral or other forms of PrEP and HIV tests Which pharmacy personnel would implement intervention Comfort with prescribing PrEP and providing HIV testing Existing facilities within or near the pharmacy that provide HIV testing and counseling
Community leader	Key informant interview	6	Inclusion criteria Aged ≥18 years Work with MoHCC^g^, the City of Harare pharmacy, or organizations that may be impacted by the intervention	Seek reactions on main insights from in-depth interviews Invite input on the proposed pharmacy-based approach Identify potential implementation barriers Facilitating roles they can assume
KP program	Formal meeting	—^h^	Inclusion criteria KP program staff at the front line of outreach	Formal appointments Referral procedure preferences Tracking continuation and missed appointments

^a^SW: sexual worker.

^b^KP: key populations.

^c^PrEP: pre-exposure prophylaxis.

^d^Pharmacy owners and staff.

^e^Not limited to SWs enrolled in the KP program.

^f^FSW: female sex worker.

^g^MoHCC: Ministry of Health and Child Care.

^h^Not applicable.

#### Qualitative Studies

Trained social science researchers will conduct qualitative interviews to solicit insights that can be applied to strengthen the PrEP-in-pharmacy intervention (eg, how and where HIV testing should be conducted, how to identify study participants, and confidentiality and privacy). In-depth interviews (IDIs) will be held with SWs and pharmacy personnel, and key informant interviews will be held with community leaders and policy makers using guided questions summarized in Table 1. All interviews will be audio recorded, but in instances where individuals want to be interviewed but prefer not to be recorded, written note-taking methods will be applied. Analytic notes will be written and summarized for synthesis of information into a report that will be presented to the advisory board and stakeholders. Written informed consent will be obtained from all qualitative study interview participants. A rapid qualitative analysis approach will be used to interpret interviews in real time to facilitate intervention finalization [45]. Taken together, data from IDIs and key informant interviews and formal discussions with advisory board members will be used to develop the PrEP-in-pharmacy intervention protocol.

#### Development of the PrEP-in-Pharmacy Intervention

A workshop including the SW and pharmacy advisory boards, and pharmacists from pharmacies that qualify to implement the PrEP-in-pharmacy intervention, will be held to finalize the intervention protocol. Key outputs will be a detailed intervention protocol that has a (1) feasible implementation plan that has been endorsed by advisory boards; (2) training component for pharmacy personnel on HIV testing, PrEP, stigma reduction, and the referral procedures; (3) training component for KP program staff on how the strategy will be integrated with usual services and a referral plan acceptable to pharmacists and KP program staff; and (4) final structure for the escalating incentive (gift vouchers).

### Aim 2: PrEP-in-Pharmacy Intervention Pilot

#### Overview

**Figure 3:**
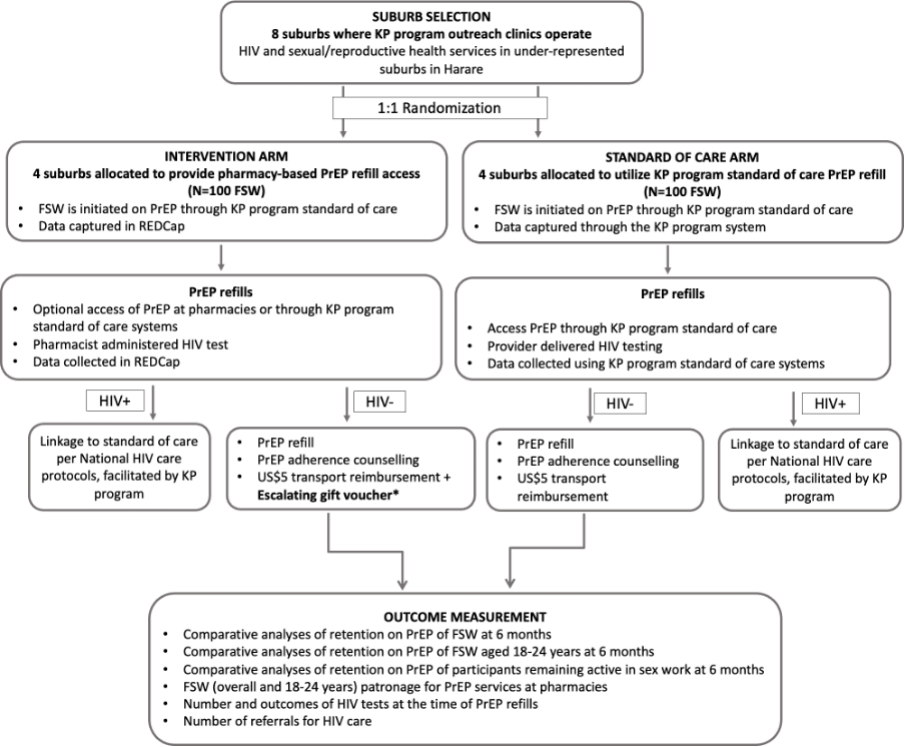
Overview of the pre-exposure prophylaxis (PrEP) in pharmacy intervention implementation strategy. FSW: female sex worker; KP: key populations; REDCap: Research Electronic Data Capture.*Escalating gift voucher structure: month 1 refill=US $5 voucher, month 3 refill=US $6 voucher, and month 6 refill=US $7 voucher.

A 2-arm pragmatic cluster-randomized trial design will be used to determine whether the addition of pharmacies as an optional PrEP refill collection point increases retention on PrEP among SWs compared to the standard-of-care services offered in the KP program (Figure 3). A 1:1 cluster randomization process will be conducted by a statistician using covariate-constrained randomization [46] in R software (version 4.1.3; R Foundation for Statistical Computing). A public randomization event will be held in which the Community Pharmacists Council, MoHCC, and other key stakeholders will participate. There will be no masking at the cluster level (suburbs), and SWs will be informed of the intervention. SWs newly enrolled in the standard-of-care suburbs will not be explicitly informed about the intervention. This trial is not blinded.

#### Overview of PrEP Services in the KP Program

**Figure 4:**
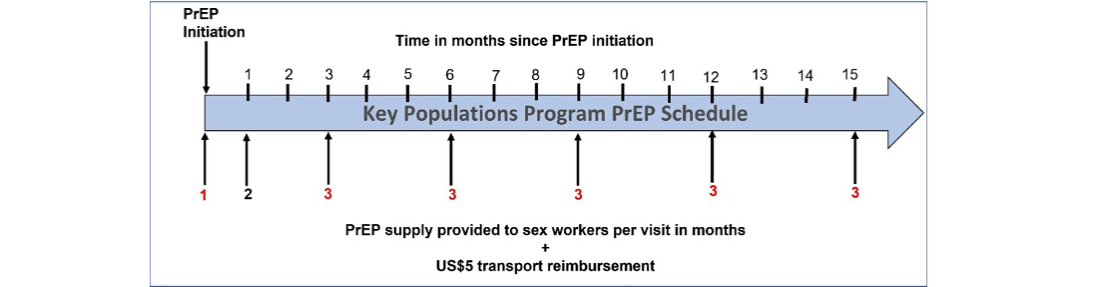
The pre-exposure prophylaxis (PrEP) refill schedule used by the key populations (KP) program, in alignment with the Zimbabwe national PrEP refill algorithm. Each PrEP refill is supplied with a US $5 transport reimbursement. Refill months indicated in red highlight where an HIV test is required to receive PrEP.

The KP program requires verbal consent from SWs to dispense PrEP, which is freely available [5]. MoHCC implementation guidelines include free provider-delivered oral rapid HIV testing at initiation, along with provision of a 1-month supply of PrEP. The first refill provides a 2-month supply of PrEP, followed by 3-month supplies for subsequent refills (Figure 4). Free, provider-delivered oral rapid HIV testing will be offered every 3 months, and in accordance with the national PrEP guidelines, only individuals with confirmed HIV-negative results are offered future PrEP refills [47]. KP program data are collected on paper-based forms for all SWs who initiate PrEP routinely; these are used to generate a unique identification number for the SWs and an associated KP program file, which is essential for PrEP refill collection. People who do not return for refill visits are routinely followed up via phone calls, WhatsApp (Meta Platforms, Inc) reminders, and peer outreach. SWs are offered a US $5 transport reimbursement when collecting their PrEP refills routinely in the program.

#### Procedures for the Standard-of-Care Arm

SWs enrolled as part of the TOPAZ study in the standard-of-care arm will initiate PrEP following the KP program standard operating procedures. They will be recruited through the KP program outreach clinics; undergo HIV testing; and if they are HIV negative, they provide verbal consent for PrEP initiation and collection of subsequent refills. At each visit, SWs will receive PrEP plus counseling on the importance of adhering to the prescribed PrEP schedule. PrEP refills will be recorded on standardized paper-based data collection tools, per the KP program standards, and will be analyzed from the program records.

Inclusion criteria for enrollment into the TOPAZ standard-of-care arm are (1) initiated on PrEP through the KP program’s mobile outreach clinics operating in 1 of the 4 standard-of-care suburbs, (2) aged >16 years, (3) having exchanged sex for money in the last 6 months, and (4) providing verbal consent.

All participants will receive a US $5 transport reimbursement at the end of each visit. Participants in the standard-of-care arm will be advised that they can collect PrEP refills from the KP program clinics, following standard procedures. Participation in the intervention will continue for 12 months.

#### Procedure for Those Enrolled Through the PrEP-in-Pharmacy Intervention Arm

FSWs initiated on PrEP through the KP program’s mobile outreach clinics in the intervention suburbs will be informed about the intervention, namely, the opportunity to access PrEP refills either from selected pharmacies within the suburbs or through KP program clinics. In addition, the participants will also be informed that PrEP refills in the pharmacy warrant gift vouchers for the first 6 months of the trial, and no vouchers will be available for the remaining 6 months of the trial. Intervention arm participants initiating PrEP will be given a TOPAZ referral slip, which they can redeem at any participating pharmacy or KP program clinic. The same services provided at standard-of-care sites will be available through the pharmacy, namely, HIV testing, PrEP refills, adherence counseling, linkage to additional services, and the US $5 transport reimbursement in line with the KP program care. In addition, FSWs that opt to refill PrEP-in-pharmacies will be offered an incentive in the form of a gift voucher redeemable for products in the pharmacy wherein the refill was collected. The incentive will continue until month 6 and will escalate in value at each visit. Months 1, 3, and 6 will receive gift vouchers valued at US $5, US $6, and US $7, respectively. To measure retention on PrEP without incentive, participants will not receive a gift voucher in the last 2 refill visits of the intervention, that is, months 9 and 12; however, data will be collected. Implementing pharmacies will be given a TOPAZ study handbook with standard operating procedures for the intervention. In addition, the KP program will conduct weekly support visits to participating pharmacies.

#### Recruitment of FSWs in PrEP-in-Pharmacy Intervention Suburbs

FSWs initiating PrEP will be informed that enrolling as an intervention participant is voluntary and their refusal will not interfere with the KP program standard of care, including their ability to access PrEP. FSWs will also be given information about the intervention and the associated risks in detail. Interested FSWs will be given an information flyer, and consent to participate will be obtained verbally. Information flyers will be available in the 3 main languages spoken in Zimbabwe: English, Shona, and Ndebele. The eligibility criteria to participate in the intervention arm are (1) initiated on PrEP through the KP program’s mobile outreach clinics in 1 of the 4 intervention suburbs, (2) aged >16 years, (3) having exchanged sex for money in the last 6 months, and (4) providing verbal consent to enroll in the TOPAZ study.

#### Data Collection for the PrEP-in-Pharmacy Intervention Arm

Data for the intervention participants will be captured onto a mobile health tool, REDCap (Research Electronic Data Capture; Vanderbilt University) [48], operating on tablets. Built-in eligibility parameters, refill scheduling, and dispensing information, as well as prompts to guide pharmacists and KP program staff through the PrEP refill process, will be programmed. To protect the privacy and confidentiality of the FSWs, at each refill, only data that pertain to the specific refill will be visible to the pharmacists through the refill interface.

#### Pharmacists’ Enrollment to Implement the PrEP-in-Pharmacy Intervention

Pharmacies will be selected to implement the PrEP-in-pharmacy intervention, based on the following criteria, and will be notified. Inclusion criteria for pharmacies participating in the TOPAZ intervention are (1) a valid pharmacy operating license; (2) interest in administering PrEP to SWs in all their diversity, in accordance with professional standards outlined in the written agreement; and (3) the presence of a private room or cubicle within the pharmacy where the HIV testing and PrEP can be provided.

Participating pharmacies will be requested to provide written consent to participate, agreeing to ensure professional, ethical, and respectful conduct toward SWs. All pharmacy personnel that are responsible for administering prescriptions within the pharmacy will undergo a series of mandatory trainings to prepare for implementation, including protocol training and a MoHCC-certified course for HIV testing.

#### Pharmacy Refill Pickup Procedure

TOPAZ intervention participants will present the TOPAZ referral slip or their KP program number to the pharmacy, and the pharmacy personnel will usher them into the private room for study-related activities, including HIV testing and PrEP refills (if eligible). FSWs who test HIV positive will be referred to the KP program for linkage to care.

#### Sample Size and Power Calculations

Data from our previous work indicate that current PrEP retention at 6 months post-PrEP initiation is 14% among FSWs in Harare. With 8 suburbs (4 per arm) and a target enrollment of 25 SWs per intervention suburb (totaling 100 participants in the intervention arm), and assuming an ICC of 0.12, the study will have ≥80% power to detect a 36% increase in PrEP retention—from 14% in the standard-of-care arm to 50% in the intervention arm. Note that this is a pilot trial and, consequently, is only powered to detect large effect sizes. If retention is lower than expected in the comparison group (eg, 10%), we will have at least 80% power to detect a ≥34% increase in PrEP retention. Likewise, if retention is higher than expected in the comparison group (eg, 30%), we will be adequately powered at 80% to detect a ≥38% increase in PrEP retention.

#### Outcomes

##### Outcome 1: Retention on PrEP at 6 Months

This will be measured as attendance within a window of −7 to +7 days of the scheduled 6-month refill visit. Outcome 1 will be expressed as the proportion of 6-month visits completed as scheduled, overall and by arm.

##### Outcome 2: PrEP Retention at 6 Months Among FSWs Aged Between 18 and 24 Years

This will be measured as attendance within a window of −7 to +7 days of the scheduled 6-month refill visit. Outcome 2 will be expressed as the proportion of visits completed as scheduled, overall and by arm.

##### Outcome 3: Patronage for PrEP Services at Pharmacies

FSWs (overall) and FSWs (aged between 18 and 24 years) patronage for PrEP services at pharmacies, defined as the proportion of all PrEP refill pickups (1, 3, 6, and 12) in intervention suburbs occurring at pharmacies, overall and by pickup month.

#### Data Analysis for Aim 2

For retention outcomes at 1, 3, 6, 9, and 12 months, the KP program database will be used to compare the number and proportion of FSWs retained on PrEP as defined earlier by study arm. We will conduct an intent-to-treat analysis to estimate the intervention’s effect using a cluster-based permutation test applied to the individual-level binary outcome (retained on PrEP and not retained on PrEP) using the *cptest* function in the R cvcrand package (or a similar function). The permutation test will be conducted with the same covariates used during the constrained randomization process at the suburb level (expanded from the values of the cluster-level covariates, according to which cluster everyone belongs). This approach will be repeated for the other outcomes. To produce a measure of effect, a Generalized Estimation Equations model will also be constructed to generate a risk difference of PrEP retention among people initiating PrEP in intervention versus comparison suburbs and a 95% CI. In accordance with the CONSORT (Consolidated Standards of Reporting Trials) guidelines, unadjusted estimates will be reported, and the preregistered analysis plan at ClinicalTrials.gov will specify an adjusted model in the case of baseline imbalances. Overall, this analysis framework permits causal attribution.

#### Data Management

All data collected in this study will be managed and transmitted by a trained data manager. A study operation manual with data management and monitoring guidelines will be developed to ensure protection of participants’ confidentiality and minimal risks due to participation in the study.

### Aim 3: Determine the Feasibility and Scalability of the Pharmacy-Based PrEP Program

#### Overview

A mixed methods evaluation, guided by the implementation science framework by Proctor et al [49], will assess implementation outcomes and how well findings link with our conceptual model, that is, how capability, opportunity, and motivation are affected. We will use the following data collection methods: (1) IDIs with FSWs and pharmacy owners and staff, KP PrEP outreach workers, and community stakeholders; (2) direct observations at intervention pharmacies; and (3) feedback from both the SWs and the pharmacist advisory boards. Collectively, data from the pilot, IDIs, and pharmacy observations will be synthesized to determine if the approach is feasible and acceptable toward optimizing the intervention for an effectiveness study.

#### Data Collection Approaches

Multiple data sources will be triangulated to evaluate the implementation science outcomes defined by Proctor et al [49], including acceptability, adoption, appropriateness, feasibility, fidelity, and penetration (Table 2). Specifically, the following data collection approaches will be used and combined with administrative data from the existing KP program to assess the implementation outcomes.

**Table 2:** Implementation outcomes and data sources.

Outcomes and analysis levels		Available measurement
**Acceptability**		
	FSW^a^	IDIs^b^ and observation at pharmacies
	Pharmacist	over the study period trained and retained over the study period
**Adoption**		
	FSW	Number of FSW opting to refill at pharmacy versus the KP^c^ program Clinic
	Pharmacist	Number of pharmacies dispensing PrEP^d^ refills
**Appropriateness**		
	FSW	IDIs, observation at pharmacies, and advisory board feedback
	Pharmacist	IDIs, observation at pharmacies, and advisory board feedback
	Community	Donors and KP program forum interviews
	MoHCC^e^	MoHCC, City of Harare pharmacy, and health care staff IDIs
	KP program staff	KP program staff interviews
**Feasibility**		
	FSW	IDIs and advisory board feedback
	Pharmacist	IDIs and advisory board feedback
Cost		Beyond the scope of this proposal
**Fidelity**		
	Pharmacists	FSW IDIs, pharmacist interviews, and observation at pharmacies
	KP Program	Referral data, self-test kit data, and refill prescription data
**Penetration**		
	Donors and KP program	Scaled to other program sites beyond Harare
Sustainability		Beyond the scope of this proposal

^a^FSW: female sex worker.

^b^IDI: in-depth interview.

^c^KP: key populations.

^d^PrEP: pre-exposure prophylaxis.

^e^MoHCC: Ministry of Health and Child Care.

#### Overview of IDIs

A trained social scientist researcher (who is not part of the KP program) will conduct IDIs with FSWs, pharmacy staff, KP program staff, and community leaders, as summarized in Table 3. All interviews will be conducted by a single interviewer in the participant’s preferred language using topical guides (Multimedia Appendices 1-3).

**Table 3:** Summary of qualitative studies to be conducted in the implementation framework study.

Group	Interview	Interviews, n	Inclusion and exclusion parameters	Interview guide
FSW^a^	Semistructured IDI^b^	20	Inclusion criteria Aged ≥16 years Initiated on PrEP^c^ during the study period At least half of the interviewed participants should be aged between 16 and 24 years Users and nonusers of pharmacies Retained and not retained on PrEP Willing and able to provide written informed consent (or witnessed thumbprint and mark for those who cannot read and write) Exclusion criteria Too physically or mentally unwell to participate, as determined by staff observation	About their experience being part of the study and being on PrEP Why did they use or not use the pharmacy for PrEP refills If relevant, their experience going to the pharmacy for PrEP refill pickup (focusing on interactions at the shop and perceived stigma) as well as getting HIV testing or using and reporting results with the HIVST^d^ Their perspectives on the short-term incentive About how we might improve the pharmacy experience and increase general interest in pharmacy-based PrEP refill visits About how we might improve the pharmacy experience and increase general interest in pharmacy-based PrEP refill visits Other KP^e^ program services they may wish to have offered through pharmacies
Pharmacy personnel from pharmacies that were involved in the TOPAZ^f,^^g^ intervention	Semistructured IDI	24	Inclusion criteria Aged ≥18 years Own a pharmacy or work at a pharmacy in Harare Pharmacists located in the KP program suburbs	Implementation challenges and strengths Enthusiasm for the approach Reactions to catering for FSWs and distributing PrEP refills and HIV testing Opinions on the design of an expanded future program Benefits for the clientele and business’ profitability, reputation, professional self-worth, and job satisfaction Strategies for longer-term sustainability and opportunity and barriers for further market expansion
Community leader	Key informant interview	5	Inclusion criteria Aged ≥18 years Work with MoHCC^h^, the City of Harare, NAC^i^, or any organizations that may be impacted by the intervention	To assess the appropriateness of the pharmacy-based intervention Potential for scale throughout Harare and beyond
KP program	Semistructured IDI	5	Inclusion criteria Age ≥18 years Employee of the KP program (including outreach workers) in 1 of the 8 study suburbs of Harare Employee of the KP program for at least 60 days Directly involved in the initiation and retention of FSWs on PrEP	Explore the appropriateness of the pharmacy-based PrEP intervention

^a^FSW: female sex worker.

^b^IDI: in-depth interview.

^c^PrEP: pre-exposure prophylaxis.

^d^HIVST: HIV self-test.

^e^KP: key populations.

^f^TOPAZ: Together Optimizing PrEP Access in Zimbabwe.

^g^Pharmacy owners and staff.

^h^MoHCC: Ministry of Health and Child Care.

^i^NAC: National AIDS Council.

#### Direct Observations at Intervention Pharmacies

Trained social science researchers will conduct direct observation at 2 pharmacies per intervention suburb using a structured data worksheet. Pharmacy owners and staff will be informed of the plans for direct observation during the informed consent process for participation for aim 2. Direct observations will be scheduled ahead of time to coincide with times when there is an expected high number of refill visits, based on REDCap data. Pharmacies will be informed of the observation by the social scientist upon arrival. A random selection of observation date and time per pharmacy will be performed using R software.

#### Data Analysis for Aim 3

Analysis will proceed according to the rapid qualitative analysis approach described in the formative research (aim 1). Interviews will be linked to pilot study data (aim 2), including pharmacy-level data to triangulate findings on FSWs’ satisfaction with pharmacies, outcomes, and opinions solicited about the feasibility and acceptability of the program. To synthesize recommendations for refining the intervention, transcripts will be analyzed using the Proctor framework [49] as a guide, with a focus on scale-up and potential adjustments to be optimized and tested in a future effectiveness study. Results of direct observations will be analyzed to assess implementation fidelity and will also be triangulated with the findings from the IDIs.

#### Description of Indicators to Proceed or Not to Proceed

The traffic light system will be applied to determine if the intervention will progress to a hybrid implementation effectiveness trial. Indicators for implementation effectiveness will be considered using a qualitative assessment (Table 4). If all 4 indicators have a positive response, that denotes a green light to proceed. If any of the indicators show a need for changes that are modifiable and none that are not modifiable, the intervention will be assigned an orange light, while a red light is denoted by ≥1 indicators requiring changes that cannot be made.

**Table 4:** Criteria to progress to a hybrid implementation effectiveness trial.

Indicator^a^	Method of assessment
1. Was it feasible to implement the pharmacy-based intervention?	Observation of intervention delivery, program data, and process evaluation interviews
2. Was the pharmacy-based intervention acceptable to beneficiaries?	Program data, process evaluation interviews, and C-RCT^b^ outcomes
3. Was the pharmacy-based intervention acceptable to pharmacists and stakeholders?	Program data, process evaluation interviews, facilitators’ questionnaire, and C-RCT outcomes
4. Were the evaluation methods acceptable and feasible?	Completeness of program to allow outcome assessment

^a^A traffic light system was used to indicate the outcome of the decisions for the Go/No-Go criteria. Green was used as a very strong indication to proceed; all four categories had a positive response. Orange was used as a moderate indication to proceed after intervention refinement; one or more categories had opportunity for improvement. Red was used as an indication of doubt as to whether to proceed; one or more categories could not be addressed.

^b^C-RCT: cluster randomized control trial.

## Results

This study protocol is compliant to the Standard Protocol Items: Recommendations for Intervention Trials (Multimedia Appendix 4). This study began the enrollment of participants in March 2024. Data collection ended in May 2025. The expansive analysis of these data will be conducted in February 2026, after all data have been collected and reviewed.

## Discussion

### Anticipated Findings

The TOPAZ intervention aims to co-develop and implement a pharmacy-based model for enhancing access to and continuation on PrEP among FSWs, fostering a key partnership between the national KP program and the private sector. The study provides a unique opportunity to explore the role of pharmacies in HIV programs. Success of the intervention will likely pave the way for greater involvement of pharmacies in HIV testing, prevention, and treatment programs. Of note, Zimbabwe’s MoHCC is currently reviewing whether HIV self-tests can be used for supporting PrEP continuation. If this is approved, we anticipate this will further simplify the program and enhance its accessibility and feasibility. This work will be shared with the community, policy makers, and health care professionals at local and international conferences, as well as through peer-reviewed publications.

This study is limited in that we will use prescription refills and self-reported adherence as a measure for PrEP continuation. A future study can improve on this by conducting more accurate measures of adherence, for example, point-of-care urine tenofovir testing in the pharmacy setting. Another limitation of the study is that it does not differentiate the effect of voucher incentives and pharmacy collection on PrEP continuation. However, follow-up beyond 6 months (when voucher incentives stop) will provide valuable information on the impact of the intervention without the vouchers. The study is also limited by its focus on women who already access the KP program, excluding those who do not attend and may be more vulnerable. The KP program is implementing various initiatives to engage SWs who do not visit program clinics. Evidence of the effectiveness of the TOPAZ intervention may be important for informing future pharmacy-based interventions for SWs who are not part of the program. It is possible that pharmacies that participate in the study are different from those that do not. Inclusion of both a pharmacy and an SW advisory board will be useful for getting views and impressions outside participating pharmacies.

### Conclusions

This study presents a protocol for integrating pharmacies into PrEP distribution, in alignment with the WHO’s recommended differentiated service delivery approach. Privately owned pharmacies are easily accessible in the community and can be applied as a sustainable conduit for PrEP delivery, especially for SWs and other groups who could benefit from PrEP.

## Appendix

Multimedia Appendix 1 Topical guide for interviews with sex workers.

Multimedia Appendix 2 Topical guide for interviews with pharmacists.

Multimedia Appendix 3 Topical guide for key informant interviews with stakeholders.

Multimedia Appendix 4 SPIRIT (Standard Protocol Items: Recommendations for Interventional Trials) checklist.

Multimedia Appendix 5 Peer review report from HIBI - HIV/AIDS Intra- and Inter-personal Determinants and Behavioral Interventions Study Section Risk, Prevention and Health Behavior Integrated Review Group (National Institutes of Health, USA).

## Abbreviations

CONSORT: Consolidated Standards of Reporting Trials
FSW: female sex worker
IDI: in-depth interview
KP: key populations
MoHCC: Ministry of Health and Child Care
PrEP: pre-exposure prophylaxis
REDCap: Research Electronic Data Capture
SW: sex worker
TOPAZ: Together Optimizing PrEP Access in Zimbabwe

## Data Availability

Datasets will be deposited in publicly available repositories.
