# Supplementary material for: Establishing a Pharmacy-Based Pre-Exposure Prophylaxis Program for Young Women Who Sell Sex: Protocol for a Randomized Controlled Trial
Source: JMIR Res Protoc. 2025 Dec 3;14:e74141. doi: 10.2196/74141 (PMC12712568; doi:10.2196/74141)
Supplement: Multimedia Appendix 1 [file resprot_v14i1e74141_app1.pdf]

MRCZ Number: MRCZ/A/2988

The Centre for Sexual Health and HIV AIDS Research Zimbabwe (CeSHHAR Zimbabwe)
4 Bath Road, Belgravia, Harare
Harare, Zimbabwe
Phone +263 – 242-3045583,333393,3320740

Study title: Pharmacy-based PrEP for Young Women who Sell Sex in Zimbabwe

In-Depth Interview Guide – FSW, Aim 1

1.	Date:	**(DD/MM/YY):** / /
2.	Interviewer name:	
3.	Participant ID:	
4.	Participant age:	
5.	Participant sex:	
6.	Participant role at pharmacy:	
7.	Pharmacy ID:	
8.	Start time:	
9.	Finish time:	

MEDICAL RESEARCH COUNCIL OF ZIMBABWE
APPROVED
30 JAN 2023
20 CAMBRIDGE ROAD
AVONDALE, HARARE

Introduction and consent

1. ***Introduce yourself and the study.***

2. ***Obtain written informed consent [READ CONSENT FORM]***
   a. Did you ask if the participant has any questions? ☐ YES ☐ NO
   b. Did participant agree to participate? ☐ YES

☐ NO and *STOP*

c. Did you give participant a copy of consent? ☐ YES ☐ NO

3. ***Obtain permission to audio record.***

   We would like to audio-record the interview so that we can record everything we discuss here today. You can also request that I turn off the recorder at any time.

   a. Is it okay with you if I audio-record? YES *TURN ON RECORDER* ☐
      NO *TAKE NOTES* ☐

4. ***Turn on recorder and <u>say Participant ID into the recorder!</u>***

Instructions: The questions below outline main categories of questioning. The sub bullets detail topics for further probing. There is no need to ask every question, and no need to stick to this order of questions, but rather to focus on the areas in which the respondent has the most to say.

Before we start, I would like to remind you of some important things to keep in mind during our conversation. Please remember that everything about this study is completely voluntary, and you should not feel compelled to share anything you do not want to. Everything you say will be kept confidential; nothing will be shared with government officials, or anyone outside of the research team. As we are asking questions that might lead you to think about sensitive topics, if you do not like a question, or if you want to end the interview, you are free to do so. Nothing bad will happen.

A. Personal background

This section is intended to get the participant, her situation, and her aspirations. Long answers are not needed, but try to get a basic understanding of where her SRH priorities fit into her life.

First, I'd like to get to know you a little better.

Can you tell me a bit about yourself? (***To ease into the interview, ask the participant about some basic information, such as what she does for fun, how many people she has in her family, her school grade and/or what she studies, etc.***)

Can you tell me about the people who are most important to you in your daily life? ***Probes***:

- What do you do when you spend time together?
- How often do you see one another? Talk to one another?
- Tell me about a time when he/she influenced a decision that was important to you.
- Is there anyone else that you may see a little less often but who is important to you? (***If yes, probe with previous questions.***)

What are some of the things that are going well for you in your life right now that make you happy?

What are some of the things that are more difficult? ***Probes***:

- What do you worry about?
- What would you like to be different about your life one year from now?

B. Experiences with PrEP and HIV prevention services

Please tell me a little bit about your experience receiving HIV prevention services with the Sisters program. What do you like about it? What do you not like about it?

What are some of the challenges you experience in obtaining the services from the Sisters program?

Pre-exposure prophylaxis (PrEP) is medicine that is taken to prevent a person who is at risk of getting HIV from getting it. Before this discussion, had you ever heard of PrEP? What do you know about it? Have you ever used PrEP? Do you know people who are currently using PrEP? What do they say about it?

If currently taking PrEP or ever used PrEP: What was/is your experience like using PrEP? If you stopped, why did you stop? What are some of the challenges in staying on PrEP? Why do you think some women start it and then do not stay on it?

Apart from the Sisters program, where else in the community can you get PrEP or other HIV prevention services? Why do you choose the Sisters program instead of these other options?

New PrEP technologies are also on the horizon. These include long-acting injectable PrEP and a vaginal ring. Have you heard about these products?
If no, describe in more detail

Would you be interested in using either of these newer PrEP technologies? Why or why not? What seems appealing or less appealing about these?

Would you be interested in having access to these newer PrEP technologies at the pharmacy? Why or why not?

C. Experiences with pharmacies

Tell me about the last time you visited a pharmacy. (***If she does not go to drug shops, skip to section I.***) ***Probe*s:**

- Tell me more about that.
- What types of items did you buy? How come?
- Why did you choose to go to that pharmacy instead of another place?
- Did you encounter any challenges in getting what you needed? (***Probe***: Where did you get the money?)
- Tell me about the other people in the pharmacy while you were there.
    - How did they make you feel? Did they affect anything you wanted to do?
    - How did this compare to other times you have visited a pharmacy in the past few months? Are there often other customers there, or do you have privacy?
- What time of day did you go? Why did you go then?
- Who was with you? Tell me more about that.
- Is this similar to other times in the last month that you've visited a pharmacy?

How often do you visit pharmacies? (***Probe*:** How many times have you visited in the last month? In the last two weeks?)

What do you go to a pharmacy to get? How come? ***Probes*:**

- How easy is it to pay for those items? How do you get the money to buy these items? How much money do you usually spend?
- What products do you love? Are there any products that you wish you could buy but never have? Tell me more.
- Tell me about the last time it was a hard choice to decide which item to buy. How did you decide?
- Do all pharmacies have the same types and quality of products? Do some shops have better ones? Tell me more about that. Do you ever get these products anywhere else?

Do you have a specific pharmacy you usually go to? Why do you go to that one?

Tell me about the people who work there. ***Probes:***

- What are they like? How familiar are they with you? With your family or friends?

- How does this affect your decision to buy things there or not?

When do you usually visit a pharmacy? (***Probe time, day of thé week.)*** How come? In the last few months, did you always visit at this [***day / time***]?

Tell me about a positive experience you've had at a pharmacy in the last month. (***Probe:*** Tell me more about that.)

Tell me about the worst experience that you can remember at a pharmacy in the last few months. (***Probe*:** Tell me more about that.)

D. Experiences with/reactions to HIV self-testing kits

[HIV self-testing is where one collects their own sample, conducts the HIV test and interprets the result with or without assistance from a health worker]. These are kits that test for HIV in blood or oral fluid. I'll give you a while to look at it.

Have you ever used an HIV self-test kit?

HAND THE TEST KIT TO THE PARTICIPANT. ENCOURAGE HER TO OPEN IT AND LOOK AT WHAT IS IN THE PACKAGE WITHOUT INSTRUCTING HER ON HOW IT WORKS. WAIT UNTIL SHE HAS FINISHED LOOKING.

What do you think about this product? ***Probes:***
- Tell me more about that.
- Is there anything that you find interesting about this product?
- Is there anything that you like about this product?
- Anything you don't like or that seems confusing about this product?

Do you have any questions about this product?

DEMONSTRATE HOW THE TEST KIT WORKS. SHOW WHAT IS INCLUDED IN THE PACKAGE. AFTERWARD, PLACE IT IN FRONT OF THE PARTICIPANT.

Can you tell me more about what you think about this product? (***Probe:*** Is there anything you like? Anything that you dislike? Anything that is confusing?)

Do you have any questions about this product?

We are planning a study in which we will provide access to PrEP refills and HIV self-testing at pharmacies for female sex workers who are starting PrEP in the Sisters program.

What do you think about picking up PrEP refills at a pharmacy? What do you think you might like about this option? What are some of your concerns about this option?

In order to be eligible to get your PrEP refill, you would need to provide the pharmacist with the results of your HIV self-testing. What do you think would be the best way to do this? Would you prefer to take the test in the pharmacy? Or would you prefer to bring the results of the test to the pharmacy (e.g., return the completed test or take a picture of it with your phone)?

Please describe any thoughts or concerns you might have about using an HIV self-test to confirm your HIV status before continuing PrEP. How do you feel about giving these results

to the pharmacist?

E. Thoughts about the Gift Card

As part of the research we are planning, women who are eligible to pick up their PrEP refills at the pharmacy will also receive a $5 gift card that is redeemable at the pharmacy. Women will get a $5 gift card for filling their first refill, a $6 gift card for filling the second refill and a $7 gift card for filling the third refill.

What are your thoughts about the gift card? What do you like about the idea and what do you dislike about the idea?

Do you think this gift card will help women who want to stay on PrEP stay on PrEP? Why? What might be more helpful?

Are there products at the pharmacy that women would be interested to buy with the gift card? What kind of things do you think women would use the gift card for?

Thank you for your time. Is there anything else you would like to say or talk with me about today?

Turn off recorder, thank the participant for her time, and give her the $5.

MEDICAL RESEARCH COUNCIL OF ZIMBABWE
APPROVED
30 JAN 2023
20 CAMBRIDGE ROAD
AVONDALE, HARARE
