# Supplementary material for: Establishing a Pharmacy-Based Pre-Exposure Prophylaxis Program for Young Women Who Sell Sex: Protocol for a Randomized Controlled Trial
Source: JMIR Res Protoc. 2025 Dec 3;14:e74141. doi: 10.2196/74141 (PMC12712568; doi:10.2196/74141)
Supplement: Multimedia Appendix 2 [file resprot_v14i1e74141_app2.pdf]

MRCZ Number: MRCZ/A/2988

CeSHHAR Zimbabwe

The Centre for Sexual Health and HIV AIDS Research Zimbabwe (CeSHHAR Zimbabwe)
4 Bath Road, Belgravia, Harare
Harare, Zimbabwe
Phone +263 – 242-3045583,333393,3320740

Study title: Pharmacy-based PrEP for Young Women who Sell Sex in Zimbabwe

In-Depth Interview Guide – Pharmacy Owners and Staff, Aim 1

1.	Date:	(DD/MM/YY): / /
2.	Interviewer name:	
3.	Participant ID:	
4.	Participant age:	
5.	Participant sex:	
6.	Participant role at pharmacy:	
7.	Pharmacy ID:	
8.	Start time:	
9.	Finish time:	

MEDICAL RESEARCH COUNCIL OF ZIMBABWE
APPROVED
30 JAN 2023
20 CAMBRIDGE ROAD
AVONDALE, HARARE

Introduction and consent

1. ***Introduce yourself and the study.***

2. ***Obtain written informed consent [READ CONSENT FORM]***
   a. Did you ask if the participant has any questions? ☐ YES ☐ NO
   b. Did participant agree to participate? ☐ YES

☐ NO and *STOP*

c. Did you give participant a copy of consent? ☐ YES ☐ NO

3. ***Obtain permission to audio record.***
   We would like to audio-record the interview so that we can record everything we discuss here today. You can also request that I turn off the recorder at any time.
   a. Is it okay with you if I audio-record? YES *TURN ON RECORDER* ☐
      NO *TAKE NOTES* ☐

4. ***Turn on recorder and <u>say Participant ID into the recorder!</u>***

Instructions: The questions below outline main categories of questioning. The sub bullets detail topics for further probing. There is no need to ask every question, and no need to stick to this order of questions, but rather to focus on the areas in which the respondent has the most to say.

Before we start, I would like to remind you of some important things to keep in mind during our conversation. Please remember that everything about this study is completely voluntary, and you should not feel compelled to share anything you do not want to. Everything you say will be kept confidential; nothing will be shared with government officials, other people who work at your pharmacy, or anyone outside of the research team. As we are asking questions that might lead you to think about sensitive topics, if you do not like a question, or if you want to end the interview, you are free to do so. Nothing bad will happen.

Some of the questions ask about customers in your pharmacy. When you are answering these questions, please don't tell me the customers' names or identifying information about them. Some questions ask about young women in your community. When I say young women, I am talking about people around ages 15 to 24 years old.

A. Pharmacy operations, customers, and products

First, I'd like to learn a little bit about you and your time at this pharmacy:

How long have you [**worked at / owned**] this pharmacy? Why did you start [**working here / buy or open the pharmacy**]?

What's the best part about your work? What about it do you enjoy or makes you happy?

What do you think is the most important part of [**your work / your pharmacy**] for the community? Why is that?

Can you tell me about a time in the last few weeks that you felt proud of [**your work / your pharmacy**]? (***Probe:*** Tell me more about that.)

What's the hardest part about your work?

Can you tell me about a time in the last few weeks that you felt frustrated at work? (***Probe:*** Tell me more about that.)

Can you tell me about the last day that you worked in the pharmacy, from the beginning of your day to the end? (***Probe:*** Can you tell me about the types of customers who came in? What types of things did they ask for?)

What times is the pharmacy busiest? Why do you think that is?

What times is the pharmacy slowest? Why do you think that is?

Can you tell me more about the types of customers who come into your pharmacy? (***Probe for age, gender, frequency of customers.***) What do customers usually need your help with? ***Probes:***

- How often do the same customers come to your store? Do you have customers that you recognize or know by name when they come in?
- Do you ever stay in touch with or talk to customers outside of the pharmacy? Tell me more about that.

What kinds of customers do you prefer to help? Why is that? ***Probes***:
- Who are your best customers? How come?
- Can you tell me about a customer that came in during the last week that you were happy to have in your pharmacy? Why is that?

What kinds of customers is it frustrating or difficult to help? Why is that? ***Probes***:
- Who are your worst customers? How come?
- Can you tell me about a customer that came in during the last week that was challenging or difficult to have in your pharmacy? Why is that?

Are there ever times where a customer asks for something that your pharmacy carries but you do not want to sell it to them? Why is that? (***Probe:*** Tell me about the last time that you did not want to sell something to a customer.)

Do you ever refuse to sell something to a customer? Why is that? (***Probe:*** Tell me about the last time that you refused to sell something to a customer.)

Do you ever ask customers to leave your pharmacy? Why is that? (***Probe:*** Tell me about the last time that you asked a customer to leave your pharmacy.)

What do you do when a customer can't afford a product that they may need? Why?

Tell me about the products you sell in this pharmacy. What types of products do you sell most often? ***Probes:***
- Are there any products that you offer that you sell very rarely? Why do you think that is? Why do you continue to offer them for sale?
- Are there any products that you enjoy selling or that you think are especially important to offer at your pharmacy? Why is that?
- Are there any products that you do *not* like to sell? Why is that?

If owner: How do you decide which products to sell in this pharmacy? ***Probes:***
- How do you decide how much to sell a product for?
- Has there ever been a time when you've started carrying a new item because a customer asked for it? Tell me more.
- How do you get information about new products?
- When there's a new product available, how do you decide whether or not to stock it?

B. Views on female sex workers, young women who sell sex

Now I want to ask you some questions about women who sell sex or exchange sex for goods, and about whether they come to your pharmacy.

Do you know if young women who sell sex come to your pharmacy? How do you know?

What kinds of things do these young women come to your pharmacy for? ***Probes***:
- Do they buy these things for themselves or someone else?

- Are there products that young girls ask for that you do not carry? Would you be interested in stocking these? Why is that?
- How much money do these young women spend on average when they visit your pharmacy? Is this different for girls in school in comparison to young women who are not in school?
    - How does this compare to other customers?
    - Do you know where they get the money to buy products at your pharmacy?
- Tell me more about how they choose which products they buy.
    - Can you share a story of when someone influenced what product a woman purchased? Is that common?
    - Who are the main people who influence how they spend money?
- How similar or different is it when a girl comes in by herself compared to when she comes with someone else (e.g., friends)?

During a typical day at the pharmacy, how many women who sell sex come into the pharmacy? ***Probes*:**
- When do they usually come in?
- Do they usually come into the pharmacy alone or with someone else? Who are they with?

Is there anything you like about helping women who sell sex who come to your store? (***Probe*:** Tell me about a time in the past year when you felt proud to help a woman who sells sex in your pharmacy.)

Is there anything that you dislike or find challenging about this group? (***Probe:*** Tell me about a time in the past year when you did not want to sell something to a woman who sells sex.)

How does helping women who sell sex compare to helping other customers?

I'd like to learn more about your views on health of women in your community particularly as it relates to HIV.

Do you think that HIV is a problem that affects women? Tell me more about that. ***Probes:***
- Why or why not?
- Do you think it is common for women and young women to have HIV? What puts them at risk of getting it?

Can you tell me generally about women's and young women's access to health services like contraceptives and pregnancy tests and HIV tests in your community? ***Probes:***
- Where should a woman go if she's worried that she has HIV? What about if she wants to get contraception?
- Have you seen any changes in recent years that have increased or reduced access?
- What do you think is the biggest challenge for women's access to these services?

C. Introducing PrEP & HIV self-testing kits

[HIV self-testing is where one collects their own sample, conducts the HIV test and interprets the result with or without assistance from a health worker. Pre-exposure prophylaxis (PrEP) is medicine that is taken to prevent a person who is at risk of getting HIV from getting it].
We are planning a study for which we will enroll pharmacies to work with our team to distribute PrEP and HIV self-testing to women who sell sex in the community. Have you ever heard of PrEP? Can you describe what it is? Have you ever heard of HIV self-testing? Can

you describe what it is?

If have never heard of PrEP, describe what it is.

Do customers ever ask you about HIV self-test products? ***If yes***:
- What types of customers ask you about this?
- Can you tell me about the last time a customer asked you about HIV self-test products? (***Probe:*** What did you tell the customer?)

These are kits that test for HIV in oral fluid. I'll give you a minute to look at it.

HAND THE TEST KIT TO THE PARTICIPANT. ENCOURAGE HIM/HER TO OPEN IT AND LOOK AT WHAT IS IN THE PACKAGE WITHOUT INSTRUCTING HIM/HER ON HOW IT WORKS. WAIT UNTIL S/HE HAS FINISHED LOOKING.

What do you think about this product? Tell me more about that. (***Note nonverbal reaction as well.) Probes***:
- Is there anything that you find interesting about this product?
- Is there anything that you like about this product?
- Anything you don't like or that seems confusing about this product?

DEMONSTRATE HOW THE TEST KIT WORKS. SHOW WHAT IS INCLUDED IN THE PACKAGE.

Can you tell me more about what you think about this product?

As I mentioned, we are planning a study where pharmacies would provide PrEP refills and HIV self-testing to women who sell sex in the community.

Both the HIV self-testing and the PrEP tablets would be provided free of charge to eligible women (paid for by our organization).

As part of the study, we will also be providing women with a gift card redeemable at your pharmacy.

Now that you know a bit more about each of these, how would you feel about providing these services to women in your community? Why? (***Probe for differences between men/women, adults/adolescents.***)

Does anything make you nervous or uncomfortable about that idea?

Does anything make you excited about this idea? Do you think providing these services would help or harm your business in any way?

What do you think about the gift card idea? Are there other ways we could promote patronage at your pharmacy? Are you interested in promoting patronage among women at risk for HIV?

What challenges do you see in offering these services to women in the community? ***Probes:***
- Where would you recommend that the person conduct the HIV self test? Why?
- What are some different ways in which we can encourage people to bring their test kits back or provide their HIV self-test results to the pharmacist?
- What is the best way to ensure that someone who takes this test follows up and visits a doctor or nurse at a health facility nearby, like [***name of closest health facility***]?
