# Supplementary material for: Establishing a Pharmacy-Based Pre-Exposure Prophylaxis Program for Young Women Who Sell Sex: Protocol for a Randomized Controlled Trial
Source: JMIR Res Protoc. 2025 Dec 3;14:e74141. doi: 10.2196/74141 (PMC12712568; doi:10.2196/74141)
Supplement: Multimedia Appendix 3 [file resprot_v14i1e74141_app3.pdf]

MRCZ Number: MRCZ/A/2988

The Centre for Sexual Health and HIV AIDS Research Zimbabwe (CeSHHAR Zimbabwe)
4 Bath Road, Belgravia, Harare
Harare, Zimbabwe
Phone +263 – 242- 3045583,333393,3320740

CeSHHAR Zimbabwe

Study title: Pharmacy-based PrEP for Young Women who Sell Sex in Zimbabwe

In-Depth Interview Guide – Community Stakeholders, Aim 1

1.	Date:	**(DD/MM/YY):** / /
2.	Interviewer name:	
3.	Participant ID:	
4.	Participant age:	
5.	Participant sex:	
6.	Participant role in community:	
8.	Start time:	
9.	Finish time:	

MEDICAL RESEARCH COUNCIL OF ZIMBABWE
APPROVED
30 JAN 2023
20 CAMBRIDGE ROAD
AVONDALE, HARARE

Introduction and consent

1. ***Introduce yourself and the study.***

2. ***Obtain written informed consent [READ CONSENT FORM]***
   a. Did you ask if the participant has any questions? ☐ YES ☐ NO

   b. Did participant agree to participate? ☐ YES

   ☐ NO à *STOP*

c. Did you give participant a copy of consent? ☐ YES ☐ NO

3. Obtain permission to audio record.

We would like to audio-record the interview so that we can record everything we discuss here today. You can also request that I turn off the recorder at any time.

a. Is it okay with you if I audio-record? YES *TURN ON RECORDER* ☐
NO *TAKE NOTES* ☐

4. Turn on recorder and <u>say Participant ID into the recorder!</u>

Instructions: The questions below outline main categories of questioning. The sub bullets detail topics for further probing. There is no need to ask every question, and no need to stick to this order of questions, but rather to focus on the areas in which the respondent has the most to say.

Before we start, I would like to remind you of some important things to keep in mind during our conversation. Please remember that everything about this study is completely voluntary, and you should not feel compelled to share anything you do not want to. Everything you say will be kept confidential. As we are asking questions that might lead you to think about sensitive topics, if you do not like a question, or if you want to end the interview, you are free to do so. Nothing bad will happen.

Some questions ask about young women in your community. When I say young women, I am talking about people around ages 15 to 24 years old.

A. Personal background

Note: Select only a few of these questions as warm-up.

First, I'd like to learn a little bit about you and your role in this community. Can you tell me a bit about yourself? Can you tell me about your role in the community?

What's the best part about your role in the community? (***Probe:*** What about it do you enjoy or makes you happy?)

What do you think is the most important part of your role for the community? Why is that? Can you tell me about a time in the last few weeks that you felt proud of what you do for the community? (***Probe***: Tell me more about that.)

What's the hardest part about your role in the community? (***Probe***: What about it do you find challenging or difficult?)

Is there anything you like about helping women at risk of HIV in your community? ***Probes***:

- Tell me about a time in the past year when you felt proud to help a woman at risk of HIV in your community.

Is there anything that you dislike or find challenging about this group? (***Probe***: Tell me about a time in the past year when you found it difficult to help a woman at risk of HIV.)

Can you tell me about a time when a young woman in your community was struggling or having trouble? (***Probe about what they did in response to this situation.***)

B. Views on Access to HIV Prevention Services

I'd like to learn more about your views on services for women at risk of HIV, specifically women who sell sex or exchange sex for gifts/goods.

In general, what do you think are the biggest challenges for women who need access these services?

Where do women generally go to get these services? What kinds of barriers do they face? Why?

Pre-exposure prophylaxis (PrEP) is medicine that is taken to prevent a person who is at risk

of getting HIV from getting it. We are planning a research project where we will provide access to PrEP refills and HIV self-testing (where one takes their own sample, conducts an HIV test and interprets the results, HIV self-testing) for women initiating PrEP through the Sisters program. We'll talk more about HIV self-testing in a moment. For now, what are your thoughts on women at risk for HIV being able to obtain PrEP refills from pharmacies?

Why do you feel that way? Do you think this is how others in your community feel, too?
- What are the benefits of women being able to get PrEP refills at pharmacies?
- Is there anything negative about seeking these services at pharmacies?
- How well are women at risk for HIV treated at pharmacies? Are they treated differently than other customers?
- How affordable are products and services at pharmacies generally? What is the quality of these products and services?

How do you think pharmacies could be made to better support women at high risk of HIV, and women who sell sex? (***Probe***: Tell me more about that. Can you think of any other ways pharmacies could be made to better support women at risk for HIV?)

C. Reactions to PrEP refills & HIV self-testing kits at pharmacies

Do people in the community ever talk about HIV self-test products? ***If yes:***
- What types of people talk about this?
- Where can people in the community find HIV self-test products?

In our project, we will enroll pharmacies to provide HIVST to women who are filling PrEP refills at the pharmacy. This is only for a limited pilot basis. These are kits that test for HIV in saliva [***SHOW SAMPLE KIT***]. I'll give you a minute to look at it.

HAND THE TEST KIT TO THE PARTICIPANT. ENCOURAGE HIM/HER TO OPEN IT AND LOOK AT WHAT IS IN THE PACKAGE WITHOUT INSTRUCTING HIM/HER ON HOW IT WORKS. WAIT UNTIL S/HE HAS FINISHED LOOKING.
What do you think about this product? Tell me more about that. (***Note nonverbal reaction as well.***) ***Probes***:
- Is there anything that you find interesting about this product?
- Is there anything that you like about this product?
- Is there anything you don't like or that seems confusing about this product?

DEMONSTRATE HOW THE TEST KIT WORKS. SHOW WHAT IS INCLUDED IN THE PACKAGE.
Can you tell me more about what you think about this product?

Do you have any questions about this product? (***Probes***: What information would you want to know in order to decide if this product made sense for people in your community to use?)

Do you think people in your community would want these HIV self-test kits? Which kinds of people do you think would be most interested in this? Why? (***Probe for differences between men/women, adults/adolescents.***)

How would you feel about people in your community using this? Why? ***Probe for differences between men/women, adults/adolescents. Probes***:
- Does anything make you nervous or uncomfortable about that idea?
- Where would you recommend that people conduct the test? Why?

What challenges do you see in people in your community using these self-test kits? (***Probe***:

What could be done to address these challenges?)

What challenges do you see in making these test kits available at pharmacies for women who are picking up PrEP refills? (***Probe***: What could be done to address these challenges?)

Thank you for your time. Is there anything else you would like to discuss related to pharmacy-based PrEP refills, women at risk of HIV in your community, or the use of HIV self-testing?

Turn off recorder, thank the respondent for his/her time, and give him/her the $5.

MEDICAL RESEARCH COUNCIL OF ZIMBABWE
APPROVED
30 JAN 2023
20 CAMBRIDGE ROAD
AVONDALE, HARARE
