# Supplementary material for: Establishing a Pharmacy-Based Pre-Exposure Prophylaxis Program for Young Women Who Sell Sex: Protocol for a Randomized Controlled Trial
Source: JMIR Res Protoc. 2025 Dec 3;14:e74141. doi: 10.2196/74141 (PMC12712568; doi:10.2196/74141)
Supplement: Multimedia Appendix 5 [file resprot_v14i1e74141_app5.pdf]

SUMMARY STATEMENT
(Privileged Communication)

PROGRAM CONTACT:
Holly Campbell-Rosen
240-627-3316
holly.campbell-rosen@nih.gov

Release Date: 04/01/2022
Revised Date:

Application Number: 1 R34 MH129220-01A1
Formerly: 1R34MH129220-01

Principal Investigators (Listed Alphabetically):

MCCOY, SANDRA I
SIBANDA, EUPHEMIA LINDELWE (Contact)

Applicant Organization: CENTRE/SEXUAL HLTH/HIV AID RES/ZIMBABWE

Review Group: HIBI
HIV/AIDS Intra- and Inter-personal Determinants and Behavioral Interventions Study Section
AIDS - EXP. REV.

Meeting Date: 03/10/2022
Council: MAY 2022
Requested Start: 07/01/2022
RFA/PA: PA20-141
PCC: 9A-ASPQ

Project Title: Pharmacy-based PrEP for Young Women who Sell Sex in Zimbabwe

SRG Action: Impact Score:20 Percentile:4 +
Next Steps: Visit https://grants.nih.gov/grants/next_steps.htm
Human Subjects: 30-Human subjects involved - Certified, no SRG concerns
Animal Subjects: 10-No live vertebrate animals involved for competing appl.
Gender: 1A-Both genders, scientifically acceptable
Minority: 5A-Only foreign subjects, scientifically acceptable
Age: 1A-Children, Adults, Older Adults, scientifically acceptable

Project Year	Direct Costs Requested	Estimated Total Cost
1	178,471	289,272
2	177,549	287,777
TOTAL	356,020	577,049

ADMINISTRATIVE BUDGET NOTE: The budget shown is the requested budget and has not been adjusted to reflect any recommendations made by reviewers. If an award is planned, the costs will be calculated by Institute grants management staff based on the recommendations outlined below in the COMMITTEE BUDGET RECOMMENDATIONS section.

1R34MH129220-01A1 Sibanda, Euphemia

RESUME AND SUMMARY OF DISCUSSION: This application proposes to examine whether pharmacies can be designed to be safe spaces for PrEP refills for female sex workers in Zimbabwe. First, female sex workers, pharmacy staff and community leaders will be interviewed about PrEP distribution, procedures for HIV self-testing, and linkage to care. Then the intervention will be piloted with female sex workers in the Sisters with a Voice program, a national PrEP program for sex workers in Zimbabwe. Female sex workers will be randomly assigned by their suburb in Harare to receive the intervention (PrEP refills at three pharmacies and escalating incentives for completing refills) or to the standard Sisters with a Voice program. The primary outcome, PrEP refill pickups, will be measured at 7 months post-intervention. To understand the implementation of the intervention, additional interviews will be conducted with female sex workers, pharmacy staff/owners, community leaders, and Sisters with a Voice staff, and the provision of PrEP at the pharmacies will be observed. The prevalence rate of HIV in female sex workers in Zimbabwe rises rapidly as they reach age 24 such that 1 in 2 female sex workers have HIV. Therefore, this effort to continue the use of PrEP by this extremely high risk group of women has the potential to have a substantial public health impact. This collaboration between the Centre for Sexual Health and HIV AIDS Research and U.S.-based researchers provides the study team with excellent resources and support. The committee felt that this was an innovative approach that had the potential to be scalable and could become widely employed throughout Zimbabwe. The investigative team has been highly responsive to concerns raised in the prior review of this application. Private spaces will now be provided for HIV self-testing in the pharmacies, concerns about the escalating incentives and their sustainability were more fully addressed, and the size of the study was reduced to 12 pharmacies in 4 suburbs (N=200) with more time for recruitment and time to complete the other study aims. Other concerns that were raised by the reviewers were also addressed. As a result, the committee's overall enthusiasm for this significantly improved resubmission was very high.

DESCRIPTION (provided by applicant): Global prevalence of HIV among female sex workers (FSW) is high. In Eastern and Southern Africa 10.4% and 33.3% of FSW are HIV infected. In Zimbabwe, prevalence estimates among FSW are as high as 78% in some regions and incidence is estimated at 5-10% per annum (over 10 times that in adult women more generally), with incidence highest among younger women and those recently engaged in sex work. Modelling suggests that with high uptake and continuation pre-exposure prophylaxis (PrEP) could potentially reduce HIV incidence in FSW by 40%, critical for its own sake but also for epidemic control. Modelling also suggests that 40-80% of all new infections in sub-Saharan Africa by 2035 are likely directly or indirectly attributable to transmission as a result of selling sex. Although PrEP is highly effective and demonstration projects show that PrEP is feasible to deliver and acceptable among FSW in Africa, the majority of sex workers who initiate PrEP stop taking it within the first few months due to a mix of structural and behavioral barriers. Many of these barriers can potentially be addressed through interventions that leverage psychosocial support, behavioral economics and structural issues. We propose to co-develop and evaluate an intervention to directly tackle poor PrEP continuation through a pharmacy-based intervention designed to increase access to PrEP and reduce stigma around its use. If successful, this preliminary study will pave the way for a future effectiveness trial to evaluate this intervention more comprehensively. Using a participatory process, we will work collaboratively with pharmacy owners and FSW to design safe and convenient spaces for PrEP refill pick-ups bundled with HIV self-testing (HIVST) and a gift card incentive (Aim 1); we will then pilot the intervention among women initiating PrEP through CeSHHAR's existing Sisters with a Voice program to evaluate the impact on PrEP retention at 7 months (Aim 2); finally, using an implementation science approach, we will use mixed-methods to understand the potential for scale of the FSW-focused pharmacy program, and based on the findings, translate lessons learned into the design of a future effectiveness trial (Aim 3). The proposed study will explore whether pharmacies can be used to distribute PrEP refills among FSW. If effective the intervention could be adapted for other

PrEP users and for new PrEP technologies as they become available. The results will provide guidance on the acceptability of pharmacy-based PrEP services, bundling PrEP with HIVST and a gift card incentive, and whether pharmacy-based distribution is feasible, acceptable, and worthy of future development.

PUBLIC HEALTH RELEVANCE: Despite clear evidence that pre-exposure prophylaxis (PrEP) is a highly effective tool for HIV prevention, we have yet to optimize its use, particularly among those most at risk of HIV such as female sex workers (FSW). In this study, we will use a participatory process to co-develop and pilot a pharmacy-based PrEP distribution intervention bundled with HIV self-testing and a gift card incentive, designed to improve PrEP access and reduce stigma, and ultimately improve continuation among FSW living in Harare, Zimbabwe. The results from the study will provide guidance for a larger R01 evaluation.

CRITIQUE 1

Significance: 1
Investigator(s): 1
Innovation: 2
Approach: 3
Environment: 1

Overall Impact: This is a well-written and responsive resubmission of a proposal to develop and pilot test pharmacy-delivered PrEP as an intervention to increase retention in PrEP among female sex workers in Zimbabwe, using a cluster-randomized trial design. The team did a nice job addressing concerns in the previous application by increasing feasibility; clarification of the provision of private spaces in participating pharmacies; and improving linkages between drug shops and the FSW-tailored clinics for referrals and other services. The work is significant and the premise that drug shops may be useful at increasing access is supported by the team's prior work on HIV self-testing in drug shops and other evidence from the literature. The team is outstanding, has complementary expertise, and has collaborated extensively on previous work in Zimbabwe, including work in pharmacies and drug shops that provided the preliminary data for this study. The environment is excellent, and the applicant organization has successfully led a large number of HIV prevention and care research projects that were subsequently scaled up on a national level; there are strong letters of support from government and the retail pharmacist association. Innovation is high with use of pharmacies or drug shops in an African setting, decentralized PrEP delivery within a FSW-focused service model, the use of pharmacy gift cards as incentives, and flexibility of the model to incorporate other PrEP or family planning modalities in future. The approach is very detailed and rigorous, with a qualitative phase followed by the trial and an aim to evaluate implementation outcomes. Use of the COM-B model of behavior change and nudge theory is strong, and they include input from key stakeholders (FSW and pharmacy owners/staff) and exploration of the use of POC urine tests (possibly included). While the number of facilities in the trial is small, this work would provide evidence on feasibility and acceptability to inform a larger study and the impact on appropriate retention in PrEP care, which is currently very low. Main limitation is challenge mapping PrEP use to needs, lack of an adherence outcome if POC urine data is not collected, and limitation of not being able to disentangle the pharmacy-based refill location from the incentive used.

1. Significance:

Strengths

- Female sex workers in Zimbabwe are at high risk for HIV, yet have had low retention in PrEP programming, due at least in part to challenges with refills and the opportunity cost of clinic appointments.
- The premise that increasing access to PrEP refills in drug shops will decrease barriers to PrEP refills by increasing access, and could increase retention in care is supported by evidence from prior studies in Zimbabwe, many carried out by the investigative team
- The proposal aims to use evidence from prior work with pharmacies to refine and operationalize the program model, then test it in a randomized trial and evaluate lessons learned for potential scale-up; the premise for this study is strong
- The ability to adapt this approach to dalpivirine ring or even potentially injectable PrEP regimens is a plus
- The work has the potential to inform a larger trial scaling up the intervention in Zimbabwe, and the team has experience with scaling up other HIV services such as HIV self-testing

Weaknesses

- None noted

2. Investigator(s):

Strengths

- The team is outstanding, has complementary expertise, and has collaborated extensively on previous work in Zimbabwe, including work that provided the preliminary data for this study
- MPI Sibanda is an ESI and has led a number of studies of HIV and SRH interventions at CeSHHAR
- MPI McCoy is an epidemiologist with expertise in intervention design, including with pharmacies and drug shops
- The MPIs have worked together for 7 years and have a detailed MPI leadership plan
- Co-I Cowan helped establish the Sister's program in Zimbabwe and has helped mentor the MPI team
- Co-I Packel has expertise in the development of interventions using incentives
- Co-I Liu is a health economics with experience conducting HIV and SRH studies in African settings

Weaknesses

- None noted

3. Innovation:

Strengths

- PrEP delivery in private pharmacies or drug shops in African settings could really help with PrEP scale up, and innovative models for doing this are needed
- The proposed approach integrating the pharmacy delivery with the standard clinic and peer support provided by Sisters for other services is innovative

- Using pharmacy gift cards as incentives is also an innovation and could encourage pharmacies to offer HIV prevention products including PrEP, as they will get increased business
- The flexibility to incorporate other PrEP modalities or pair with contraception enhances the innovation of the model

Weaknesses

- Use of POC urine testing in pharmacies would potentially be very novel, if adopted – some preliminary data on whether this might be acceptable would have been nice to have

4. Approach:

Strengths

- The approach is detailed and rigorous, with clearly spelled out procedures for the three aims
- The theoretical underpinning of the study, with the COM-B model of behavior change and the behavioral economics concept of nudges provided by the escalating incentives is strong
- Aim 1 work to refine and operationalize the pharmacy-based PrEP distribution model includes FSW and pharmacy owners and staff, as well as an advisory board including community leaders, MOH leaders, and referral facility HIV services staff – this approach will capture diverse views on how to create a model that will be feasible, acceptable, destigmatizing, and conforming to any regulatory issues as needed.
- Incorporation of POC urine tests for PrEP adherence, if feasible and acceptable, could help with monitoring of adherence as well as retention, and would be important to test in the study if possible, before an efficacy trial in which adherence would be an important consideration
- A rapid qualitative analysis after the Aim 1 work will identify the most important salient themes and enable rapid movement to the Aim 2 work
- A cluster-randomized trial design seems very appropriate to pilot test the intervention and even though the number of facilities is somewhat small, testing out this design in an R34 will be very useful should the intervention prove promising enough to take to a larger efficacy trial.
- Recruitment of pharmacies follows a set procedure, and although it will be biased towards early adapters, this is necessary in the early stages of intervention development and testing
- The comparison condition is well described, as are procedures for randomization and follow-up.
- Procedures for pharmacy visits with HIVST and pharmacist counseling in a private booth are appropriate and include bundling with the incentive coupons
- Use of the preloaded Sisters Referral App will enhance uniformity of visits, enable data collection, and quality checks, as well as referrals to other services as needed
- Outcomes are appropriate and well considered, with use of a 7-month retention outcome, and the current outcomes could potentially be supplemented by an exploratory outcome of POC urine test results
- While not fully powered and focused on feasibility and acceptability, the number of women included will be enough to identify a moderate to large difference and enhances feasibility of the approach
- The aim 3 work to evaluate implementation outcomes using mixed methods and Proctor's IS framework is well considered and robust

Weaknesses

- PrEP retention is not the same as adherence, and collecting at least some data on adherence – it was not clear if self-reported adherence would be collected if POC urine tests are deemed not to be feasible or acceptable, or to map sexual behavior and pill-taking to determine if PrEP use is matching need
- The limitation of not being able to remove the pharmacy refill effect from the incentive effect is still there, but it seems that the MOH has provided incentives in the past of a similar scale, and if this is something the pharmacies like, perhaps they can be provided loyalty points or discounts in the future

5. Environment:

Strengths

- CeSHHAR has led a large number of HIV prevention and care research projects and led scale-up of interventions on a national level
- Strong letters of support are provided from CeSHHAR, the national and city MOHCC/DOH, the retail pharmacists association in support of the proposed work

Weaknesses

- None noted

Study Timeline:

Strengths

- The timeline is more feasible than what was apparently in the prior application, and seems quite reasonable, with appropriate milestones

Weaknesses

- None noted

Protections for Human Subjects:

Acceptable Risks and/or Adequate Protections

- Risks and benefits are carefully weighed, and protections are well considered

Data and Safety Monitoring Plan (Applicable for Clinical Trials Only):

Acceptable

- Comprehensive plan, with acceptable level of detail

Inclusion Plans:

- Sex/Gender: Distribution justified scientifically
- Race/Ethnicity: Distribution justified scientifically
- For NIH-Defined Phase III trials, Plans for valid design and analysis: Not applicable
- Inclusion/Exclusion Based on Age: Distribution justified scientifically

- Participants will be women (female sex workers), with race/ethnicity reflecting that of Zimbabwe, and ages 16 and up included - this is appropriate given the goal of targeting FSW, both young and old, and the disproportionate HIV risk faced by young women

Vertebrate Animals:

Not Applicable (No Vertebrate Animals)

Biohazards:

Not Applicable (No Biohazards)

Resubmission:

- The resubmission is responsive to feedback from reviewers, by decreasing the scope and increasing feasibility; clarification of the provision of private spaces in participating pharmacies, which will opt into the study; clarification that the inability to disentangle the incentive from the pharmacy PrEP provision is a limitation of the study but is potentially scalable; and improved linkages between the Sisters clinics for services beyond PrEP refills, which will take place in drug shops closer to where participants live.

Applications from Foreign Organizations:

Justified

- A foreign justification is provided, and there is a strong rationale for basing the trial in Zimbabwe.

Resource Sharing Plans:

Acceptable

- No relevant resources, however a data sharing plan has been provided.

Budget and Period of Support:

Recommend as Requested

CRITIQUE 2

Significance: 1
Investigator(s): 1
Innovation: 1
Approach: 2
Environment: 1

Overall Impact: The goal of the resubmission R34 proposal is to adapt and refine a pharmacy-based intervention for female sex workers (FSW) in Harare, Zimbabwe. The investigative team was highly responsive to the prior critiques and based on the changes to the resubmission, the proposed research is likely to have a high impact in the field. Strengths of the proposed research include the strong

scientific premise as evidenced by the investigative team and others' research highlighting the importance for innovative approaches to increase PrEP retention and adherence among FSW; the highly innovative nature of the proposal including the ability to be flexible in accounting for other PrEP modalities as they become available and approved; the strong scientific rigor demonstrated by the study design, theories, utilization of a cluster randomized design, and utilization of Advisory Boards to further adapt and refine the intervention. Finally, the investigative team has a strong presence in Zimbabwe and the proposed research naturally builds upon the investigative team's research trajectory.

1. Significance:

Strengths

- The proposed research focuses on a key population – FSW in Zimbabwe; FSW have a higher risk of HIV acquisition and this risk increases substantially with younger age.
- Although there is a high level of interest in PrEP, there are significant issues with lack of PrEP continuity among FSW, which will be the focus of the proposed intervention.
- Scientific premise is strong as evidenced by the reviewed literature and investigative team's preliminary studies that provide support for alternative intervention methods needed to increase PrEP retention and adherence among FSWs.
- Pharmacy-based interventions offer a convenient, scalable approach for PrEP distribution by pharmacists, accounting for a multitude of barriers reported by FSW (e.g., access, cost, transportation, etc.).

Weaknesses

- None noted.

2. Investigator(s):

Strengths

- mPI Sibanda is an early-stage investigator and epidemiologist with over 10 years of experience conducting research among FSWs; currently the recipient of a competitive fellowship (UKRI MRC/DFID African Research Leader Fellowship).
- mPIs Sibanda and McCoy have a longstanding history of collaborating on projects in Zimbabwe; other members of the investigative team have collaborated as evidenced by shared publications.
- The investigative team have expertise in relevant topical areas (e.g., implementation science, epidemiology, pharmacy, economics, mixed methods) to support the proposed project.
- Co-I Cowan has mentored mPI Sibanda for over a decade and is also the founder of the Sisters program.
- Strong multiple PI leadership plan in place.

Weaknesses

- None noted.

3. Innovation:

Strengths

- PrEP provision by way of private pharmacists.
- Utilization of non-monetary financial incentives to encourage FSW to pick up refills.
- Pharmacies that are in support of PrEP provision with no indication of community-level stigma will be targeted.
- The study is positioned to account for new PrEP modalities as they become available (e.g., long-acting).

Weaknesses

- None noted.

4. Approach:

Strengths

- The intervention combines the COM-B (Capability, Opportunity, and Motivation – Behavioral) model with the behavioral economic theory, applied to FSW.
- Leverage of the Sisters program as the comparison group for the cluster randomized trial design.
- An objective marker of PrEP adherence based on urine testing will be explored for feasibility and acceptability for a future R01.
- Utilization of a FSW Advisory Board and pharmacist Advisory Board (quarterly meetings), integrated into Aims 1 and 3.
- Implementation outcomes (Aim 3) will be determined through a mixed-methods evaluation guided by Proctor's implementation science framework.
- Reduction of sample size and revised timeline ensures feasibility of completing the study aims within the three year funding period.
- Methods for each study aim are comprehensive and appropriate.
- Rigor is strong given the randomization process, study design (i.e., cluster randomized trial design), and analysis plan for each aim.

Weaknesses

- Unclear whether the analysis accounts for service seeking through Sisters since participants will be able to have access to all available Sisters service regardless of study arm assignment.
- The exact training topics focused on how stigma can be prevented in the pharmacy environment are vague (i.e., not presented).
- Unclear how urine testing will be explored for feasibility for future scale up.

5. Environment:

Strengths

- Adequate resources provided at CeSHHAR, UCSF, the University of California Berkeley, and Liverpool School of Tropical Medicine to support the proposed research aims.
- CeSHHAR has had a number of collaborations with US-based academic institutions (e.g., UCSF, UC Berkeley, UNC Chapel Hill, University of Pittsburg).

- Strong letter of support provided by CeSHHAR in Zimbabwe, community partner for the proposed project.
- Additional letters of support provided by AIDS & TB Programme, City of Harare Health Department, Community Pharmacists Association, and the Liverpool School of Tropical Medicine.

Weaknesses

- None noted

Study Timeline:

Strengths

- The time allotted for Aim 1 activities is sufficient.
- The time allotted for Aim 3 activities is sufficient.

Weaknesses

- Training pharmacy owners and staff was not built into the timeline.

Protections for Human Subjects:

Acceptable Risks and/or Adequate Protections

- Appropriate protections in place for study participants.

Data and Safety Monitoring Plan (Applicable for Clinical Trials Only):

Acceptable

- Appropriate and comprehensive data and safety monitoring plan included.

Inclusion Plans:

- Sex/Gender: Distribution justified scientifically
- Race/Ethnicity: Distribution justified scientifically
- For NIH-Defined Phase III trials, Plans for valid design and analysis: Not applicable
- Inclusion/Exclusion Based on Age: Distribution justified scientifically
- The study will focus on women
- Given the geographic study location, the study participants will be Black, of African descent.
- The study will include children aged 16-17 and adults aged 18 and older.

Vertebrate Animals:

Not Applicable (No Vertebrate Animals)

Biohazards:

Not Applicable (No Biohazards)

Resubmission:

- The investigative team was highly responsive to the previous critiques.
- Sample size reduction and timeline adjustment ensures a more feasible project within the 3-year period.

Applications from Foreign Organizations:

Justified

- Adequate justification is provided given that the focus of the project is to target female sex workers in Zimbabwe for HIV prevention in light of the high HIV prevalence in this population.
- The Zimbabwe Ministry of Health is in the early stages of rolling out PrEP nationally, to make it free to at-risk populations.
- The proposed research leverages existing PrEP outreach services provided by Sisters with a Voice to FSW in Harare.

Resource Sharing Plans:

Acceptable

Budget and Period of Support:

Recommend as Requested

CRITIQUE 3

Significance: 2
Investigator(s): 1
Innovation: 1
Approach: 3
Environment: 1

Overall Impact: This is a resubmission that seeks to develop and pilot a multi-component pharmacy-based PrEP distribution strategy to increase FSW motivation, access, and effective use of PrEP and improve prep continuation (including HIV self-testing) and circumvent stigma and other barriers. Overall, the proposal has been highly responsive to prior critiques, is well thought through with strong scientific rationale/justification for the myriad of decisions made, and has potential for high impact. There were some minor concerns focusing on limited information about data analyses, limited data being collected about fidelity, but the major strengths of the application outweigh these minor and addressable concerns.

1. Significance:

Strengths

- Different models for PrEP distribution are needed and the proposed strategy addresses a key gap

- As retention in PrEP care is very poor, this strategy has potential for a large impact to significantly enhance PrEP persistence.
- This model is likely scalable and potentially sustainable given that pharmacies are widely accessible in LMICs
- Potential to reduce PrEP related stigma and experienced stigma towards FSW in healthcare settings

Weaknesses

- Unable to disentangle effects of “non-monetary” incentives and pharmacy-based delivery – although may be the subject of a future larger trial

2. Investigator(s):

Strengths

- MPI Dr. Sibanda (ESI) and Dr. McCoy are well experienced to conduct this study
- Strong team with complementary expertise including behavioral economics
- Inclusion of Ministry of Health advisors is a strength

Weaknesses

- None needed

3. Innovation:

Strengths

- New mode for PrEP roll-out in Zimbabwe
- Behavioral economics based intervention to increase health behaviors/habit formation
- Leverages pharmacies where FSW already likely frequent

Weaknesses

- None noted by reviewer

4. Approach:

Strengths

- While a pilot trial, use of a rigorous implementation science evaluation framework
- Cluster RCT design of 8 suburbs
- Well integrated theories to guide intervention

Weaknesses

- While the proposal refers to the incentive as non-monetary, a gift card is still essentially a monetary incentive.
- Unclear motivation for pharmacy staff to use tablet, conduct/offer HIVST, and collect/enter data on behalf of the study or with fidelity. Limited information about how fidelity will be measured rigorously and evaluated

- Appears to be a missed opportunity to understand/explore characteristics associated with retention in the pharmacy-based arm. Also ICC may likely be included in models for adherence/retention.

5. Environment:

Strengths

- Strong environments to conduct this study

Weaknesses

- None noted

Study Timeline:

Strengths

- Appropriate

Weaknesses

- None noted by reviewer

Protections for Human Subjects:

Acceptable Risks and/or Adequate Protections

Data and Safety Monitoring Plan (Applicable for Clinical Trials Only):

Acceptable

Inclusion Plans:

- Sex/Gender: Distribution justified scientifically
- Race/Ethnicity: Distribution justified scientifically
- For NIH-Defined Phase III trials, Plans for valid design and analysis: Not applicable
- Inclusion/Exclusion Based on Age: Distribution justified scientifically

Vertebrate Animals:

Not Applicable (No Vertebrate Animals)

Biohazards:

Not Applicable (No Biohazards)

Resubmission:

- Responsive to prior critiques

Applications from Foreign Organizations:

Justified

- justified reasons for conducting this study abroad, by a foreign organization

Resource Sharing Plans:
Acceptable

Budget and Period of Support:
Recommend as Requested

THE FOLLOWING SECTIONS WERE PREPARED BY THE SCIENTIFIC REVIEW OFFICER TO SUMMARIZE THE OUTCOME OF DISCUSSIONS OF THE REVIEW COMMITTEE, OR REVIEWERS' WRITTEN CRITIQUES, ON THE FOLLOWING ISSUES:

PROTECTION OF HUMAN SUBJECTS: ACCEPTABLE

INCLUSION OF WOMEN PLAN: ACCEPTABLE

INCLUSION OF MINORITIES PLAN: ACCEPTABLE

INCLUSION ACROSS THE LIFESPAN: ACCEPTABLE

COMMITTEE BUDGET RECOMMENDATIONS: The budget was recommended as requested.

Footnotes for 1 R34 MH129220-01A1; PI Name: Sibanda, Euphemia Lindelwe

+ Derived from the range of percentile values calculated for the study section that reviewed this application.

NIH has modified its policy regarding the receipt of resubmissions (amended applications).See Guide Notice NOT-OD-18-197 at https://grants.nih.gov/grants/guide/notice-files/NOT-OD-18-197.html. The impact/priority score is calculated after discussion of an application by averaging the overall scores (1-9) given by all voting reviewers on the committee and multiplying by 10. The criterion scores are submitted prior to the meeting by the individual reviewers assigned to an application, and are not discussed specifically at the review meeting or calculated into the overall impact score. Some applications also receive a percentile ranking. For details on the review process, see http://grants.nih.gov/grants/peer_review_process.htm#scoring.

MEETING ROSTER

HIV/AIDS Intra- and Inter-personal Determinants and Behavioral Interventions Study Section
Risk, Prevention and Health Behavior Integrated Review Group
CENTER FOR SCIENTIFIC REVIEW
HIBI
03/10/2022 - 03/11/2022

Notice of NIH Policy to All Applicants: Meeting rosters are provided for information purposes only. Applicant investigators and institutional officials must not communicate directly with study section members about an application before or after the review. Failure to observe this policy will create a serious breach of integrity in the peer review process, and may lead to actions outlined in NOT-OD-14-073 at https://grants.nih.gov/grants/guide/notice-files/NOT-OD-14-073.html, NOT-OD-15-106 at https://grants.nih.gov/grants/guide/notice-files/NOT-OD-15-106.html, and NOT-OD-18-115 at https://grants.nih.gov/grants/guide/notice-files/NOT-OD-18-115.html, including removal of the application from immediate review.

CHAIRPERSON(S)

KIPKE, MICHELE D, PHD
PROFESSOR
DEPARTMENTS OF PEDIATRICS
AND PREVENTIVE MEDICINE
KECK SCHOOL OF MEDICINE
UNIVERSITY OF SOUTHERN CALIFORNIA
LOS ANGELES, CA 90028

MEMBERS

BUTLER, LISA MICHELLE, PHD
ASSOCIATE RESEARCH PROFESSOR
INSTITUTE FOR COLLABORATION ON HEALTH,
INTERVENTION, AND POLICY
UNIVERSITY OF CONNECTICUT
STORRS, CT 06269

COMULADA, WARREN SCOTT, DRPH
ASSOCIATE PROFESSOR
DEPARTMENT OF PSYCHIATRY
AND BIOBEHAVIORAL SCIENCES
SCHOOL OF PUBLIC HEALTH
UNIVERSITY OF CALIFORNIA, LOS ANGELES
LOS ANGELES, CA 90024

DODGE, BRIAN MARK, PHD
PROFESSOR
DEPARTMENT OF APPLIED HEALTH SCIENCE
INDIANA UNIVERSITY SCHOOL OF PUBLIC HEALTH
BLOOMINGTON, IN 47405

FRIEDMAN, MACKEY R, PHD *
ASSISTANT PROFESSOR
DEPARTMENT OF INFECTIOUS DISEASES AND
MICROBIOLOGY
UNIVERSITY OF PITTSBURGH
PITTSBURGH, PA 15213

GILBERTSON, ADAM L, PHD *
ASSOCIATE RESEARCH SCIENTIST
PACIFIC INSTITUTE FOR RESEARCH AND EVALUATION
CHAPEL HILL, NC 27514

GOLUB, JONATHAN E, MPH, PHD *
ASSOCIATE PROFESSOR
CENTER FOR TUBERCULOSIS RESEARCH
SCHOOL OF MEDICINE
JOHNS HOPKINS UNIVERSITY
BALTIMORE, MD 21231

GRAHAM, SUSAN MARIE, MD, PHD
PROFESSOR
DEPARTMENTS OF MEDICINE AND GLOBAL HEALTH
SCHOOL OF MEDICINE
UNIVERSITY OF WASHINGTON
SEATTLE, WA 98104

GROV, CHRISTIAN, PHD
PROFESSOR AND CHAIR
DEPARTMENT OF COMMUNITY HEALTH
AND SOCIAL SCIENCES
SCHOOL OF PUBLIC HEALTH AND HEALTH POLICY
CITY UNIVERSITY OF NEW YORK
NEW YORK, NY 10027

HAMPANDA, KAREN MARIE, MPH, PHD *
ASSISTANT PROFESSOR
DEPARTMENT OF OBSTETRICS AND GYNECOLOGY
UNIVERSITY OF COLORADO ANSCHUTZ MEDICAL CAMPUS
DENVER, CO 80045

HANSEN, NATHAN B, PHD
DEPARTMENT HEAD AND PROFESSOR
DEPARTMENT OF HEALTH PROMOTION AND BEHAVIOR
COLLEGE OF PUBLIC HEALTH
UNIVERSITY OF GEORGIA
ATHENS, GA 30602

HEADS, ANGELA, PHD *
ASSOCIATE PROFESSOR
DEPARTMENT OF PSYCHIATRY AND BEHAVIORAL SCIENCES
UNIVERSITY OF TEXAS HEALTH SCIENCES CENTER AT HOUSTON
HOUSTON, TX 77054

HORVATH, KEITH JOSEPH, PHD
ASSOCIATE PROFESSOR
DEPARTMENT OF CLINICAL PSYCHOLOGY
SAN DIEGO STATE UNIVERSITY
SAN DIEGO, CA 92120

IWELUNMOR, JULIET, PHD
ASSOCIATE PROFESSOR
DEPARTMENT OF BEHAVIORAL SCIENCE AND HEALTH EDUCATION
COLLEGE FOR PUBLIC HEALTH AND SOCIAL JUSTICE
ST. LOUIS UNIVERSITY
ST. LOUIS, MO 63104

KIM, MARIA HYOUN, MD *
ASSOCIATE PROFESSOR
DEPARTMENT OF PEDIATRICS
BAYLOR COLLEGE OF MEDICINE
HOUSTON, TX 77030

LOVEJOY, TRAVIS IAN, PHD
ASSOCIATE PROFESSOR
DEPARTMENT OF PSYCHIATRY
SCHOOL OF MEDICINE
OREGON HEALTH AND SCIENCE UNIVERSITY
PORTLAND, OR 97239

MACDONELL, KAREN KOLMODIN, PHD *
ASSOCIATE PROFESSOR
DEPARTMENT OF FAMILY MEDICINE AND PUBLIC HEALTH SCIENCES
SCHOOL OF MEDICINE
WAYNE STATE UNIVERSITY
DETROIT, MI 48202

MACKESY-AMITI, MARY ELLEN, PHD *
ASSOCIATE PROFESSOR
DIVISION OF EPIDEMIOLOGY AND BIOSTATISTICS
SCHOOL OF PUBLIC HEALTH
UNIVERSITY OF ILLINOIS, CHICAGO
CHICAGO, IL 60453

PATEL, VIRAJ V, MPH, MD *
ASSOCIATE PROFESSOR OF MEDICINE
DEPARTMENT OF MEDICINE
ALBERT EINSTEIN COLLEGE OF MEDICINE
BRONX, NY 10461

RAMSEY, SUSAN E, PHD
ASSOCIATE PROFESSOR
DIVISION OF GENERAL INTERNAL MEDICINE
RHODE ISLAND HOSPITAL
BROWN UNIVERSITY
PROVIDENCE, RI 02903

ROTH, ALEXIS MARIE, MPH, PHD *
ASSOCIATE PROFESSOR
DEPARTMENT OF COMMUNITY HEALTH & PREVENTION
DORNSIFE SCHOOL OF PUBLIC HEALTH
DREXEL UNIVERSITY
PHILADELPHIA, PA 19104

SAFREN, STEVEN A, PHD
PROFESSOR
DEPARTMENT OF PSYCHOLOGY
COLLEGE OF ARTS AND SCIENCES
UNIVERSITY OF MIAMI
CORAL GABLES, FL 33124

SSEWAMALA, FRED M, PHD
PROFESSOR
INSTITUTE FOR PUBLIC HEALTH
BROWN SCHOOL
WASHINGTON UNIVERSITY
ST. LOUIS, MO 63130

STATON, MICHELE, PHD *
PROFESSOR
DEPARTMENT OF BEHAVIORAL SCIENCES
COLLEGE OF MEDICINE
UNIVERSITY OF KENTUCKY
LEXINGTON, KY 40536

STOCKMAN, JAMILA KINSHASA, PHD
PROFESSOR
DIVISION OF GLOBAL PUBLIC HEALTH
DEPARTMENT OF MEDICINE
SCHOOL OF MEDICINE
UNIVERSITY OF CALIFORNIA, SAN DIEGO
LA JOLLA, CA 92093

SULLIVAN, PATRICK SEAN, PHD
PROFESSOR
DEPARTMENT OF EPIDEMIOLOGY
ROLLINS SCHOOL OF PUBLIC HEALTH
EMORY UNIVERSITY
ATLANTA, GA 30322

TANNER, AMANDA E, MPH, PHD *
ASSOCIATE PROFESSOR
DEPARTMENT OF PUBLIC HEALTH EDUCATION
SCHOOL OF HEALTH AND HUMAN SCIENCES
UNIVERSITY OF NORTH CAROLINA GREENSBORO
GREENSBORO, NC 27402

THAMES, APRIL D, PHD
ASSOCIATE PROFESSOR
DEPARTMENT OF PSYCHIATRY AND BEHAVIORAL
SCIENCES
UNIVERSITY OF CALIFORNIA, LOS ANGELES
LOS ANGELES, CA 90095

TURAN, JANET M, PHD
PROFESSOR
DEPARTMENT OF HEALTH CARE ORGANIZATION
AND POLICY
SCHOOL OF PUBLIC HEALTH
UNIVERSITY OF ALABAMA AT BIRMINGHAM
BIRMINGHAM, AL 35294

WEBEL, ALLISON R, PHD
PROFESSOR
SCHOOL OF NURSING
UNIVERSITY OF WASHINGTON
SEATTLE, WA 98195

WILTON, LEO, PHD
PROFESSOR
DEPARTMENT OF HUMAN DEVELOPMENT
COLLEGE OF COMMUNITY AND PUBLIC AFFAIRS
BINGHAMTON UNIVERSITY
BINGHAMTON, NY 13902

WINDSOR, LILIANE CAMBRAIA, PHD
ASSOCIATE PROFESSOR
SCHOOL OF SOCIAL WORK
THE UNIVERSITY OF ILLINOIS AT URBANA-CHAMPAIGN
URBANA, IL 61801

SCIENTIFIC REVIEW OFFICER

RUBERT, MARK P, PHD
SCIENTIFIC REVIEW OFFICER
CENTER FOR SCIENTIFIC REVIEW
NATIONAL INSTITUTES OF HEALTH
BETHESDA, MD 20892

EXTRAMURAL SUPPORT ASSISTANT

CAMBRELEN, AMY ANGELA
EXTRAMURAL SUPPORT ASSISTANT
CENTER FOR SCIENTIFIC REVIEW
NATIONAL INSTITUTE OF HEALTH
BETHESDA, MD 20892

* Temporary Member. For grant applications, temporary members may participate in the entire meeting or may review only selected applications as needed.

Consultants are required to absent themselves from the room during the review of any application if their presence would constitute or appear to constitute a conflict of interest.
